# Functional Analysis of Pepper F-box Protein CaDIF1 and Its Interacting Partner CaDIS1: Modulation of ABA Signaling and Drought Stress Response

**DOI:** 10.3389/fpls.2019.01365

**Published:** 2019-10-30

**Authors:** Junsub Lim, Chae Woo Lim, Sung Chul Lee

**Affiliations:** Department of Life Science (BK21 Program), Chung-Ang University, Seoul, South Korea

**Keywords:** abscisic acid, F-box protein, drought, pepper, stomata, virus-induced gene silencing

## Abstract

Plant adaptive responses to environmental stress are coordinated by inhibition of plant growth and development. Abscisic acid (ABA) is a major phytohormone that regulates the stress response, and its sensitivity determines stress tolerance levels. In this study, we report the identification and functional role of a novel F-box protein, CaDIF1 (*Capsicum annuum* Drought-Induced F-box Protein 1). The expression of *CaDIF1* in pepper leaves was induced by ABA, drought, H_2_O_2_, and NaCl treatments. In comparison with wild-type pepper plants, *CaDIF1*-silenced pepper plants exhibited a drought-sensitive phenotype, whereas *CaDIF1*-overexpressing (OX) plants exhibited ABA-sensitive and drought-tolerant phenotypes. Using a yeast two-hybrid assay, we identified CaDIS1 (*C. annuum* DIF1-Interacting SKP 1), which interacts with CaDIF1 in the cytoplasm and nucleus. Consistent with *CaDIF1*-silenced pepper plants, *CaDIS1*-silenced pepper plants displayed ABA insensitivity and reduced drought tolerance, and these were characterized by larger stomatal apertures and greater transpirational water loss. Taken together, our results indicate that CaDIF1 and its interacting partner CaDIS1 synergistically regulate the ABA-dependent defence signaling response to drought stress.

## Introduction

Plants are sessile organisms; hence, they have evolved defence mechanisms to adapt to abiotic stresses, such as drought, high salinity, and extreme temperatures. When plants encounter drought stress, they modify their cellular processes to trigger the defence response, including abscisic acid (ABA) biosynthesis and reprogramming of gene expression (Lee and Luan, 2012; Lim et al., 2015a). The plant hormone ABA plays a critical role in numerous aspects of cellular processes, including plant growth, development, and adaptation to abiotic stress (Cutler et al., 2010; Lim et al., 2015a). ABA modulates defence responses to drought stress through stress-responsive gene expression, regulations of stomatal opening/closing, and root hydraulic conductivity (Sirichandra et al., 2009; Lim et al., 2015b). Under stress conditions, a large number of genes are upregulated in vegetative tissues and several plant hormones are associated with modulation of stress-related gene expression. ABA also modulates numerous genes compared with other major plant hormones (Goda et al., 2008; Mizuno and Yamashino, 2008). The induction of ABA-regulated genes, including transcription factors and E3 ligases, plays a key role in conferring enhanced stress tolerance and protecting plants from drought stress.

Protein degradation process through the ubiquitin/26S proteasome (UPS) pathway is an important mechanism involved in regulating defence responses to biotic and abiotic stresses because of its ability to rapidly remove unnecessary intracellular proteins (Lim et al., 2017). Ubiquitination is a type of post-translational modification found in eukaryotic cells, and it involves the sequential action of three key enzymes—ubiquitin-activating enzyme (E1), ubiquitin-conjugating enzyme (E2), and ubiquitin ligase (E3) (Ciechanover and Schwartz, 1998). Initially, E1 activates ubiquitin in an ATP-dependent manner and transfers activated ubiquitin to E2. Finally, ubiquitin is attached to the target protein—this process is facilitated by E3 ligase. Polyubiquitinated target proteins are degraded by the 26S proteasome system. In this process, E3 ligase defines substrate specificity and recruits target proteins. More than 1400 E3 ligases have been identified in the *Arabidopsis* genome (Vierstra, 2009; Sadanandom et al., 2012). E3 ligases are classified into two types based on their subunit compositions and are subsequently divided according to distinct functional motifs. The RING (really interesting new gene), PUB (plant U-box), and HECT (homology to E6-AP C-terminus) E3 ligases consist of a single subunit, whereas the SCF [Skp (S-phase kinase-associated protein)/cullin/F-box] and CUL4-DDB1 (CULLIN4-damaged-specific DNA binding protein1) ligases consist of a multi-subunit (Stone et al., 2005; Pazhouhandeh et al., 2011; Irigoyen et al., 2014; Seo et al., 2014).

The SCF complex is composed of Skp1, CULLIN1, a RING finger protein Rbx1/Hrt/Roc1, and F-box protein (Deshaies, 1999; Kipreos and Pagano, 2000). Among these, the F-box protein determines and delivers target protein to the complex, because F-box is associated with protein–protein interaction (Kipreos and Pagano, 2000; Koops et al., 2011). The *Arabidopsis* genome encodes 694 F-box proteins that are components of the SCF complex (Gagne et al., 2002). The SCF complex comprises the largest family of E3 ubiquitin ligases, and these ligases are associated with many processes involving signal transductions, including biotic and abiotic stress responses. Recently, overexpression of *GmSK1* conferred enhanced tolerance to high salinity and drought stress (Chen et al., 2018). SCF type E3 ligase RIFP1 negatively regulates ABA signaling and drought stress response by involvement of ubiquitination and degradation of RCAR3 ABA receptor at the nucleus (Li et al., 2016). Moreover, CRL4 type E3 ligase complex was found to degrade nuclear ABA receptor PYL8 (Irigoyen et al., 2014). The biological role of SCF type E3 ligases in defence response to drought stress through the ABA-signaling pathway has been largely investigated in several monocot and dicot plants; however, their exact role remains unclear.

To gain further insight into the molecular process of drought stress response, we isolated the F-box protein *CaDIF1* gene and the expression level of *CaDIF1* was induced in response to abiotic stress applications. Based on these expression levels of *CaDIF1* gene, we examined the biological roles in responses to ABA and drought stress treatments by using virus-induced gene-silenced pepper and overexpressed (OX) *Arabidopsis thaliana* plants. To better investigate the further biological role of CaDIF1, we performed a yeast two-hybrid analysis to identify CaDIS1, which form a complex with CaDIF1. Consistent with *CaDIF1*-silenced pepper plants, *CaDIS1*-silenced pepper plants showed ABA signaling-mediated drought-sensitive phenotype. This study suggests that CaDIF1 and CaDIS1 function together as positive modulators of ABA signaling and the drought stress response.

## Results

### Identification of the *CaDIF1* Gene and Amino Acid Sequence Analysis of the CaDIF1 Protein

**Figure 1:**
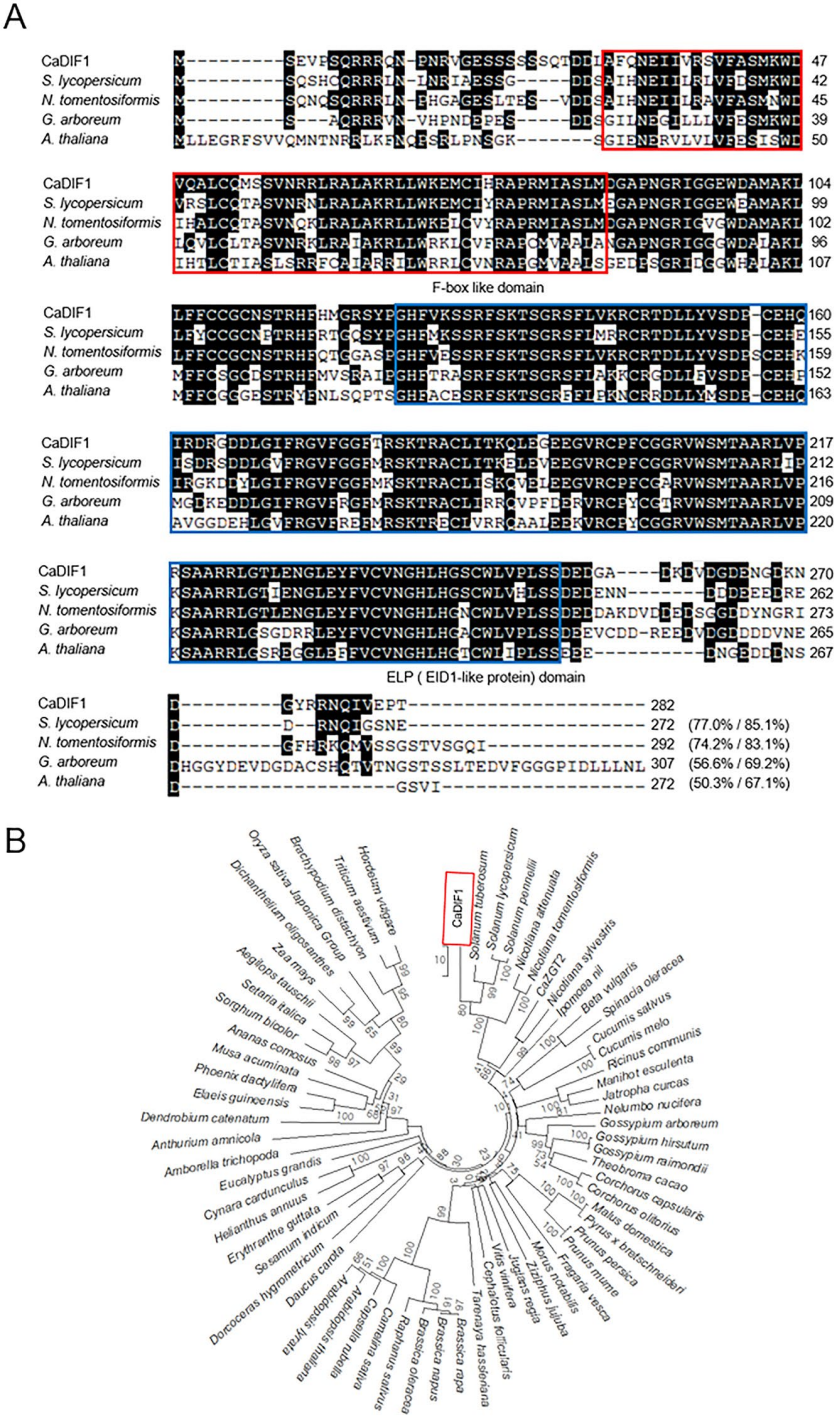
Homology of the pepper CaDIF1 (*Capsicum annuum* Drought-Induced F-box protein 1) protein. **(A)** Alignment of the deduced amino acid sequences of CaDIF1 with its homologous proteins was performed using ClustalW2. Identical and similar amino acid residues are shaded according to the percentage identity in ClustalW2. Sequences include CaDIF1 (accession no. XP_016553423.1), *Solanum lycopersicum* (accession no. XP_004247146.1), *Nicotiana tomentosiformis* (accession no. XP_009606180.1), *Gossypium arboretum* (accession no. XP_017614523.1), and *Arabidopsis thaliana* (accession no. NP_567137.1). The red box indicates the F-box like protein domain; the blue box indicates the ELP (EID1-Like Protein) domain. **(B)** Phylogenetic tree analysis of the CaDIF1 protein. BLAST search was performed using deduced amino acid sequences of CaDIF1, and sequences with the highest similarity were gathered from each plant species. Multiple sequence alignment was performed using Clustal Omega (http://www.ebi.ac.uk/Tools/msa/clustalo/) with default settings. The phylogenetic tree was drawn using the neighbour-joining method with MEGA software (version 7.0) (Kumar et al., 2016). Bootstrap values were calculated from 1000 bootstrap replications and were indicated at each branch point. Scale bar indicates genetic distance.

To isolate a drought stress inducible gene, we performed RNA-seq assay and identified the pepper *CaDIF1* gene from drought stress applied pepper leaves (Lim and Lee, 2016). The CaDIF1 consists of 282 amino acid, which are encoded by an 849 bp coding region (**Figure 1A**). The isoelectric point and molecular mass of CaDIF1 protein was 8.03 and 31,548 Da, respectively. The putative CaDIF1 protein contains highly conserved F-box and EID1-Like Protein (ELP) domains, including amino acid residues 30–86 and 125-252, respectively (**Figure 1A**). The amino acid sequence analysis of CaDIF1 by multiple alignment (**Figure 1A**) and phylogenetic tree analysis (**Figure 1B**) revealed that this protein has high homology with F-box proteins from other plant species (**Figures 1A, B**). In particular, CaDIF1 shares 50.3–77.0% identities and 67.1–85.1% similarities with other F-box proteins.

### Induction of the *CaDIF1* Gene and Subcellular Localization of the CaDIF1 Protein

**Figure 2:**
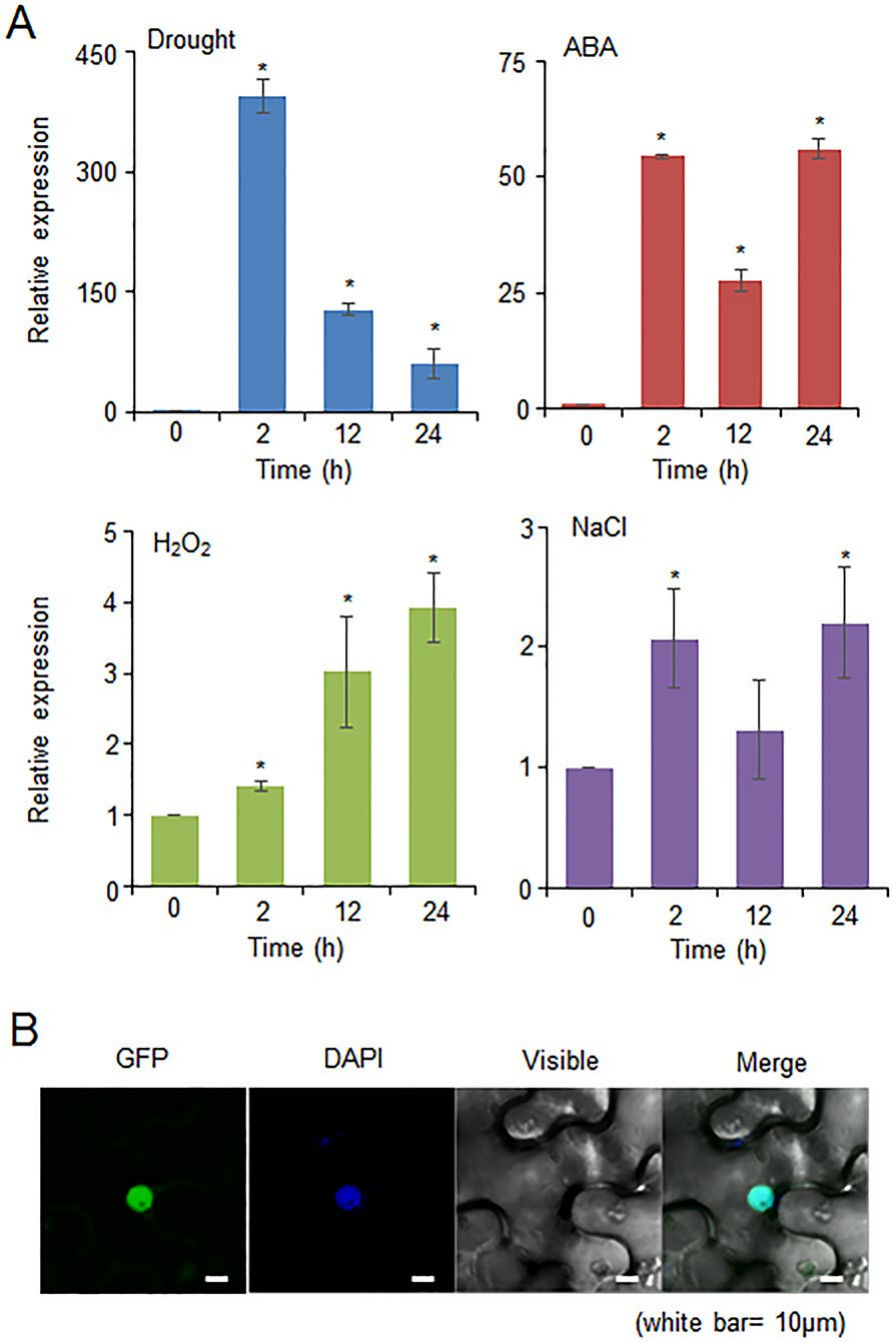
Expression of the *CaDIF1* gene and localization of the CaDIF1 protein. **(A)** Induction of *CaDIF1* in pepper leaves at various time points after treatment with drought, 100 μM abscisic acid (ABA), H_2_O_2_ (100 μM), and NaCl (200 mM). The pepper *Actin1* gene was used as an internal control. Data represent the mean ± standard error of five independent biological replicates. Asterisks indicate significant differences between 0 h and other times (Student’s *t*-test; *P* 0.05). **(B)** Subcellular localization of the CaDIF1 protein using transient expression of the green fluorescent protein (GFP) fusion protein in *Nicotiana benthamiana* cells. The 35S:*CaDIF1-GFP* construct was expressed using agroinfiltration of *N. benthamiana* leaves and observed under a confocal laser-scanning microscope. 4′,6-Diamidino-2-phenylindole (DAPI) staining was used as a marker for the nucleus. White bar = 10 μm.

The *CaDIF1* gene was identified in drought stress-treated pepper plants; therefore, we investigated whether the expression level of *CaDIF1* was increased by drought, ABA, H_2_O_2_, and NaCl treatments (**Figure 2A**). First, we investigated the expression level of *CaDIF1* transcripts in pepper leaves at different time points after drought stress treatment. The *CaDIF1* transcripts dramatically induced at all times measured. ABA is known to function as a key modulator in the defence response to water deficit condition (Jakab et al., 2005). To examine whether ABA is also associated with *CaDIF1* expression, we treated pepper plants with ABA and monitored accumulation of *CaDIF1* transcripts. The *CaDIF1* transcripts were strongly accumulated after ABA treatment. H_2_O_2_ functions as a signal component in ABA-mediated stomatal closure (Zhang et al., 2001); hence, we determined the expression level of *CaDIF1* after H_2_O_2_ treatment. The induction of *CaDIF1* transcripts were detected at 2 h after treatment and gradually increased until 24 h. High salinity treatment induced weak accumulation of *CaDIF1* transcripts.

To determine the subcellular localization of CaDIF1 protein in the plant cell, GFP-tagged CaDIF1 fusion protein was transiently expressed in the epidermal cells of *Nicotiana benthamiana*. GFP signals were generated in the nucleus (**Figure 2B**). We also detected that the blue signals localized to the nucleus overlapped with the GFP signals by using diamidino-2-phenylindole (DAPI) staining. These results suggest that the CaDIF1 has a functional role in the nucleus.

### ABA Hyposensitivity and Enhanced Drought Sensitivity of *CaDIF1*-Silenced Pepper Plants

**Figure 3:**
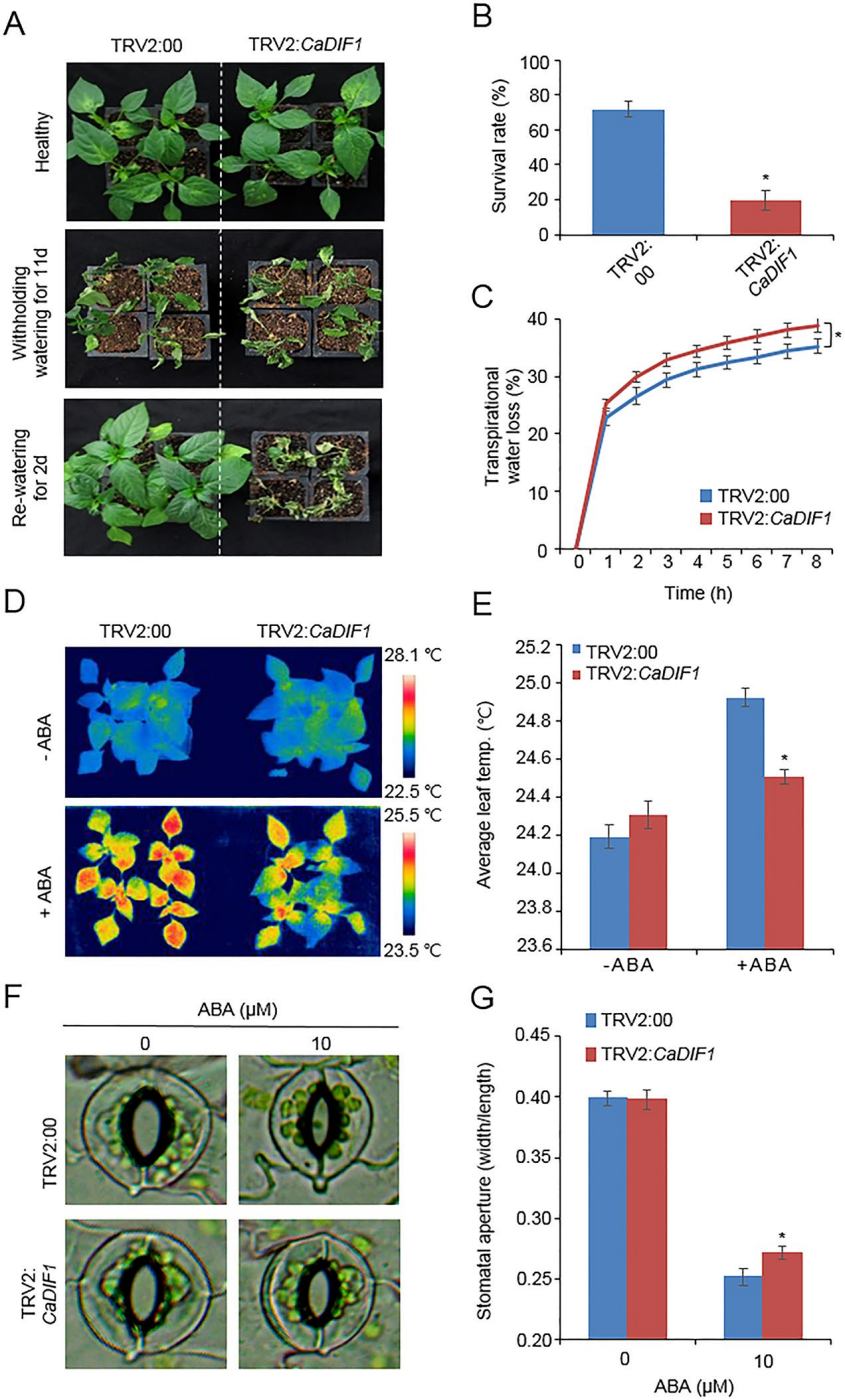
Reduced tolerance of *CaDIF1*-silenced pepper plants to drought stress. **(A)** Drought susceptibility of *CaDIF1*-silenced pepper plants. Four-week-old TRV2:*CaDIF1* and TRV2:00 pepper plants were subjected to drought stress by withholding watering for 11 days. Representative images were taken before (left) and after (middle) drought stress and 2 days after re-watering (right). **(B)** The survival rate was measured by counting the number of plants with green and rehydrated leaves 2 days after re-watering. Data represent the mean ± standard error of three independent biological replicates, each evaluating 30 plants. **(C)** Transpirational water loss from the leaves of empty vector control and *CaDIF1*-silenced pepper plants at various times after detachment of leaves. Data represent the mean ± standard deviation of three independent biological replicates. **(D** and **E)** Leaf temperatures of empty vector control and *CaDIF1*-silenced pepper plants after treatment with 100 μM ABA. Representative thermographic images were taken 6 h after treatment **(D)** and the mean leaf temperature was measured using 10 plants of each line **(E)**. Data represent the mean ± SD of three independent biological replicates. **(F** and **G)** Stomatal apertures in empty vector control and *CaDIF1*-silenced pepper plants after ABA treatment. Representative images were taken under a microscope **(F)** and the stomatal apertures were measured **(G)**. Leaf peels were harvested from 2-week-old pepper plants and incubated in stomatal opening solution containing 10 μM ABA; the stomatal apertures were then measured under a microscope. Representative images were taken before (left) and 2.5 h after (right) ABA treatment. Data represent the mean ± standard error of three independent biological replicates, each evaluating 20 plants. Asterisks indicate significant differences between the control and the *CaDIF1*-silenced pepper plants (Student’s *t*-test; *P* 0.05).

*CaDIF1* was induced by drought and ABA treatment; therefore, we postulated that this gene is involved in drought response and ABA signaling. To confirm this prediction, we conducted phenotypic analysis using loss-of function and gain-of function mutants by virus-induced gene silencing (VIGS) in pepper and generating transgenic *Arabidopsis* plants, respectively. We monitored *CaDIF1* silencing by measurement of *CaDIF1* transcripts in control (TRV2:00) and *CaDIF1*-knock down pepper (TRV2:*CaDIF1*) plants. The accumulation of *CaDIF1* transcripts was low in the *CaDIF1*-knock down pepper leaves compared with control leaves (**Supplementary Figure S1B**). We subjected control and *CaDIF1*-knock down pepper plants to drought stress and compared their phenotypes (**Figure 3A**). We withheld water (11 days; middle panel) and resupply water (2 days; right panel) and assessed growth status in both plants. After treatment of drought stress and re-watering, leaf wilting phenotypes were more appeared in *CaDIF1*-knock down pepper plants than in control plants. The survival ratios of *CaDIF1*-knock down pepper plants (19.3%) were much lower than control pepper plants (71.8%) (**Figure 3B**). Previous studies suggested that water retention capacity is associated with drought tolerance; hence, the drought-sensitive phenotype of *CaDIF1*-knock down pepper plants is probably caused by altered water retention capacity. To asses this prediction, we monitored the fresh weight of leaves detached from control and *CaDIF1*-knock down pepper plants (**Figure 3C**). The leaf fresh weight of *CaDIF1*-knock down pepper plants was low compared with that of control plants. To examine whether the difference in water retention capacity was derived from a variation of ABA sensitivity, we measured leaf temperatures (**Figures 3D, E**). Leaf temperature shows the strength of water evaporation by transpiration, with a low left temperature reflecting high level of transpiration (Park et al., 2015). In the ABA untreated condition, leaf temperatures were not significantly different between control pepper and *CaDIF1*-knock down pepper plants (**Figures 3D, E**). However, after ABA treatment, the leaf temperatures of *CaDIF1*-knock down pepper plants were significantly low compared with control pepper plants. Consistent with leaf temperatures, the stomatal apertures were significantly larger in *CaDIF1*-silenced pepper plants than in control plants (**Figures 3F, G**). These results indicate that the increased transpirational water loss in *CaDIF1*-knock down pepper plants, which leads to reduced drought tolerance, is derived from ABA hyposensitivity.

### Enhanced ABA Sensitivity of *CaDIF1*-OX Plants

**Figure 4:**
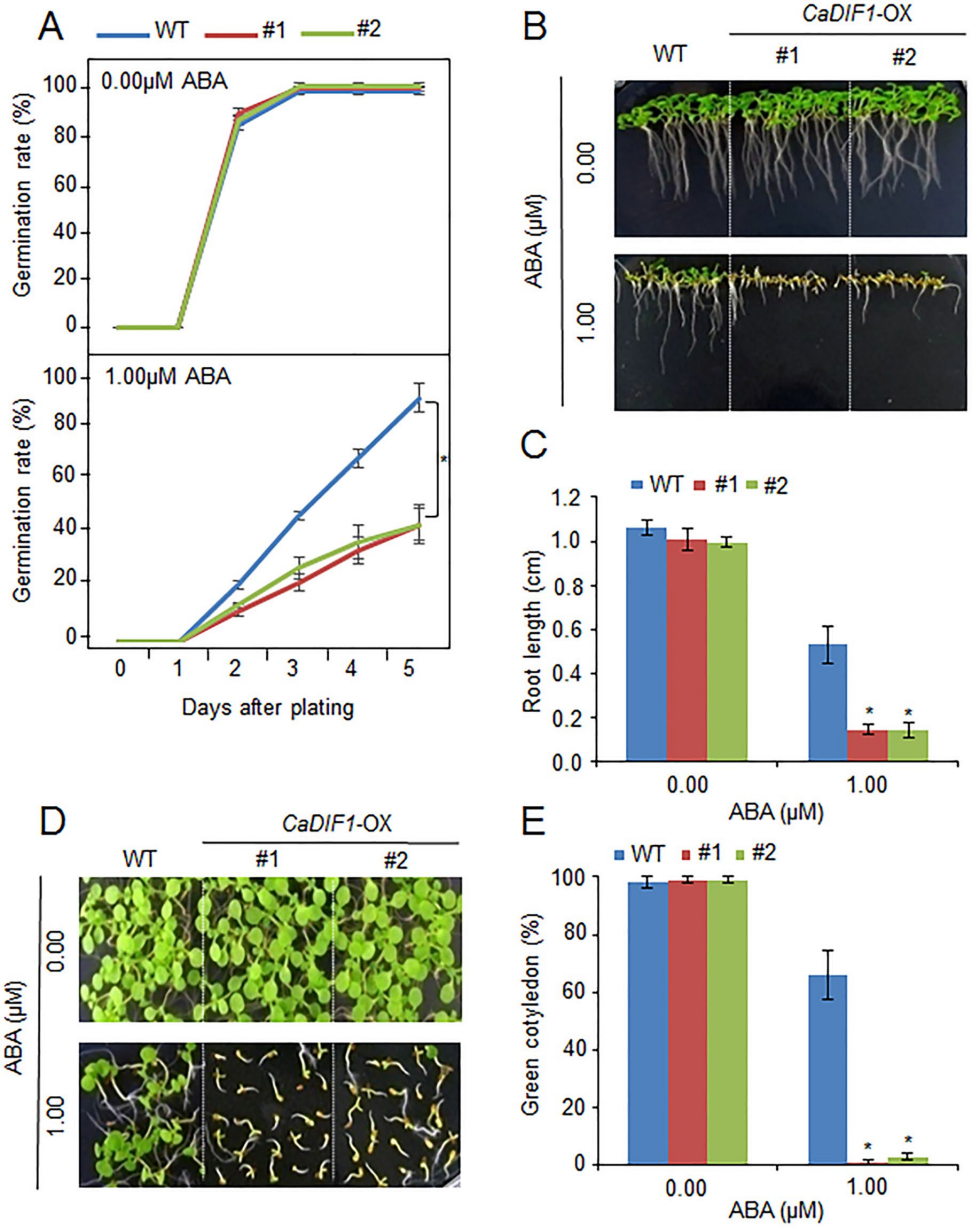
Enhanced sensitivity of *CaDIF1*-OX transgenic *Arabidopsis* lines to ABA. **(A)** Germination rates of *CaDIF1*-OX mutants and wild-type (WT) plants on 0.5× Murashige and Skoog (MS) medium supplemented with 1.0 μM ABA. **(B** and **C)** Primary root elongation of WT and transgenic lines exposed to ABA. Representative images were taken **(B)** and the root length of each plant was measured 8 days after sowing **(C)**. Data represent the mean ± standard error of three independent biological replicates, each evaluating 25 seeds. Asterisks indicate significant differences between WT and transgenic lines (Student’s *t*-test; *P* 0.05). **(D** and **E)** Seedling development of *CaDIF1*-OX mutants and WT plants exposed to ABA. Representative images were taken 7 days after plating **(D)** and the number of seedlings in each line with expanded cotyledons was recorded **(E)**. Data represent the mean ± standard error of four independent biological replicates, each evaluating 25 seeds. Asterisks indicate significant differences between WT and transgenic lines. (Student’s *t*-test; *P* 0.05).

**Figure 5:**
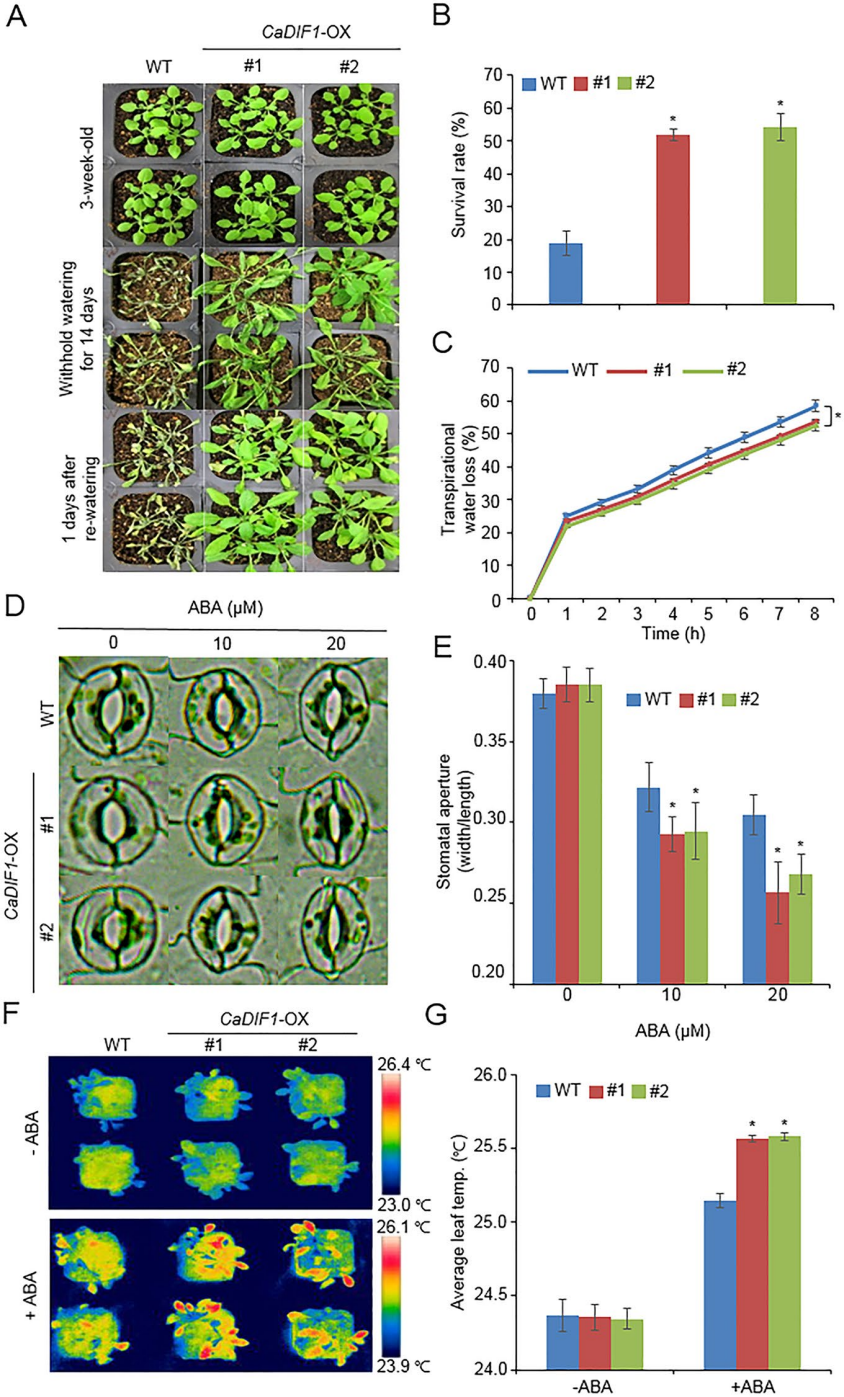
Enhanced tolerance of *CaDIF1*-OX transgenic *Arabidopsis* lines to drought stress. **(A)** Drought tolerance of *CaDIF1*-OX transgenic plants. Three-week-old WT and transgenic plants were subjected to drought stress by withholding watering for 14 days and re-watering for 1 day. **(B)** Survival rates of plants after re-watering. Data represent the mean ± standard error of three independent biological replicates, each evaluating 30 plants. **(C)** Transpirational water loss from the leaves of WT and transgenic plants at various time points after leaf detachment. Data represent the mean ± standard error of three independent biological replicates, each evaluating 50 leaves. **(D** and **E)** Stomatal apertures in WT and *CaDIF1*-OX plants treated with ABA. Leaf peels were harvested from 3-week-old plants of each line and incubated in stomatal opening solution containing 10 or 20 μM ABA. Representative images were taken under a microscope **(D)** and stomatal apertures were measured **(E)**. Data represent the mean ± standard error of three independent biological replicates. **(F** and **G)** Representative thermographic images of *CaDIF1*-OX transgenic *Arabidopsis* lines and WT plants 6 h after treatment with 100 μM ABA **(F)**; the mean leaf temperatures of the three largest leaves were measured using 10 plants of each line **(G)**. Data represent the mean ± SD of three independent biological replicates. Asterisks indicate significant differences between WT and transgenic lines (Student’s *t*-test; *P* 0.05).

To further investigate the *in vivo* function of *CaDIF1*, we generated transgenic *Arabidopsis* plants, exhibiting overexpression of the *CaDIF1* gene. A real-time transcription PCR (RT-PCR) assay revealed that the accumulation of *CaDIF1* transcripts was high in *CaDIF1*-OX plants, but not detected in the wild-type plants (**Supplementary Figure S1B**); hence, *CaDIF1*-OX plants were used in our subsequent phenotypic analysis. Under normal growth conditions, we observed no phenotypic differences between the wild-type and *CaDIF1*-OX plants (**Figures 4** and **5**). To investigate whether altered expression of *CaDIF1* affects the ABA response, we examined germination rate, primary root growth, and seedling establishment in the presence and absence of ABA (**Figure 4**). *CaDIF1*-OX seeds were germinated on MS medium supplemented with 0.0 and 1.0 µM ABA. In the presence of ABA, the germination rate of *CaDIF1*-OX seeds at 2 days after sowing was significantly lower than that of wild-type seeds (**Figure 4A**). We measured the primary root lengths in wild-type and *CaDIF1*-OX plants at 8 days after sowing (**Figures 4B, C**). In the absence of ABA, the root lengths did not differ significantly between wild-type and transgenic plants; however, in the presence of 1.0 µM ABA, the roots of *CaDIF1*-OX plants were significantly shorter than those of wild-type plants (**Figures 4B, C**). Consistent with root growth, seedling establishment of *CaDIF1*-OX plants was significantly lower than that of wild-type plants (**Figures 4D, E**). These data suggest that altered *CaDIF1* expression affects ABA response in germination and post-germination stages.

### Enhanced Drought Tolerance of *CaDIF1*-OX Plants

*CaDIF1* was markedly induced by drought stress treatment (**Figure 2A**), and *CaDIF1*-knock down pepper plants showed an altered phenotype to drought stress (**Figure 3**). Hence, we examined the *in vivo* role of *CaDIF1* in response to drought stress (**Figure 5**). First, we investigated the drought phenotype of *CaDIF1*-OX plants by withholding water for 14 days and then re-watering for 1 day (**Figure 5A**). Under well-watered conditions, we were not able to observe any phenotypic differences between *CaDIF1*-OX plants and wild-type plants (**Figure 5A**, upper panel). However, under conditions of drought stress, *CaDIF1*-OX plants exhibited a less wilted phenotype than wild-type plants (**Figure 5A**, middle panel). Furthermore, upon re-watering, the *CaDIF1*-OX plants resumed their growth rapidly compared with the wild-type plants (**Figure 5A**, lower panel). At 1 day after re-watering, the survival ratio of *CaDIF1*-OX plants was 51.8–54.2%, whereas only 18.8% of wild-type plants resumed their growth (**Figure 5B**).

To find why *CaDIF1*-OX plants had drought tolerance phenotype, we measured the transpirational water loss, leaf temperature, stomatal aperture, and stress-related gene expressions. We monitored transpirational water loss by measuring the fresh weights of detached rosette leaves (**Figure 5C**). We found that the transpirational water loss was significantly lower in *CaDIF1*-OX plants than in the wild-type plants, suggesting that the enhanced drought tolerance of *CaDIF1*-OX plants was derived from a reduced transpiration rate. To determine whether low transpirational water loss in *CaDIF1*-OX plants was caused by increased ABA sensitivity, we measured stomatal aperture and leaf temperature after treatment with various concentrations of ABA. First, we estimated significant differences in stomatal pore sizes between wild-type and *CaDIF1*-OX plants after ABA treatment (**Figures 5D, E**). ABA treatment triggered a reduction in the stomatal aperture in wild-type and *CaDIF1*-OX plants; however, transgenic plants displayed smaller stomatal apertures than wild-type plants. These results indicate that low transpirational water loss in *CaDIF1*-OX plants was derived from enhanced ABA sensitivity, especially in the stomata. Next, we measured the leaf temperatures of wild-type and *CaDIF1*-OX plants. Consistent with the stomatal aperture, *CaDIF1*-OX plants exhibited significantly higher leaf temperatures than wild-type plants (**Figures 5F, G**).

**Figure 6:**
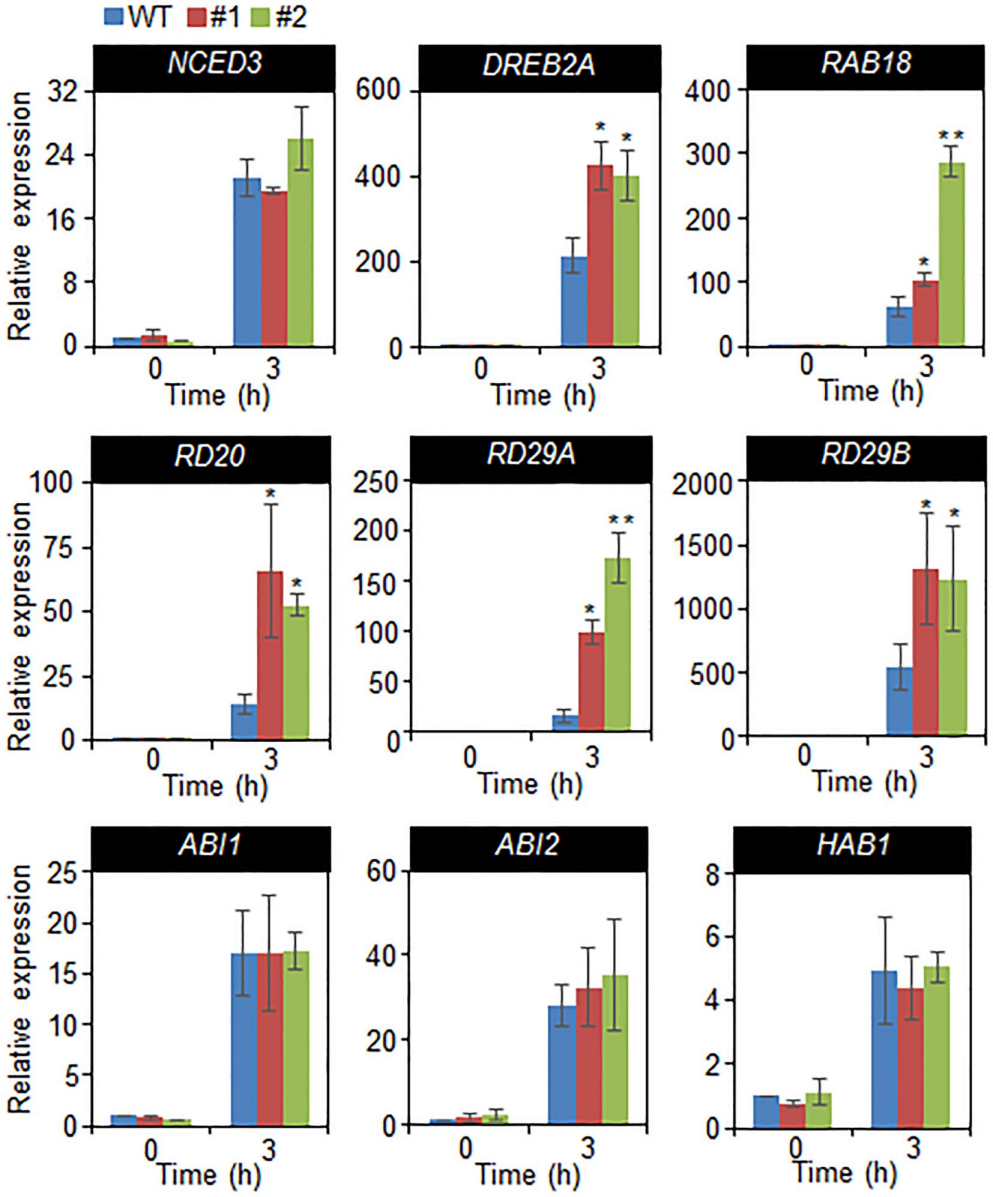
Drought-inducible genes in *CaDIF1*-OX plants. Quantitative reverse transcription PCR analysis was performed on the detached leaves of plants exposed to drought stress at 3 h after detachment. The relative expression levels (∆∆CT) of each gene were normalized to the geometric mean of *Actin8* as an internal control gene. Data represent the mean ± standard error of three independent biological replicates. Asterisks indicate significant differences between WT and transgenic lines (Student’s *t*-test; *P* 0.05).

In a previous studies, drought sensitivity and tolerance is related with the transcripts accumulation of stress-related genes, and that increased or decreased drought tolerance are related with altered expression of these genes (Cheong et al., 2007; Joo et al., 2019). Hence, we performed quantitative RT-PCR analysis of stress-related genes, including *NCED3*, *DREB2A*, *RAB18, RD20*, *RD29A*, and *RD29B*, and group A 2C type protein phosphatases (PP2Cs), such as *ABI1*, *ABI2*, and *HAB1* (**Figure 6**). The transcripts accumulation of stress-related genes were significantly high in *CaDIF1*-OX plants compared with wild-type plants. However, the expression level of 2C type protein phosphatases are not different between both plants. Collectively, these data indicate that enhanced expression of *CaDIF1* increases drought tolerance by reducing stomatal pore size and enhancing stress-related gene expression.

### CaDIF1 Interacts With CaDIS1

**Figure 7:**
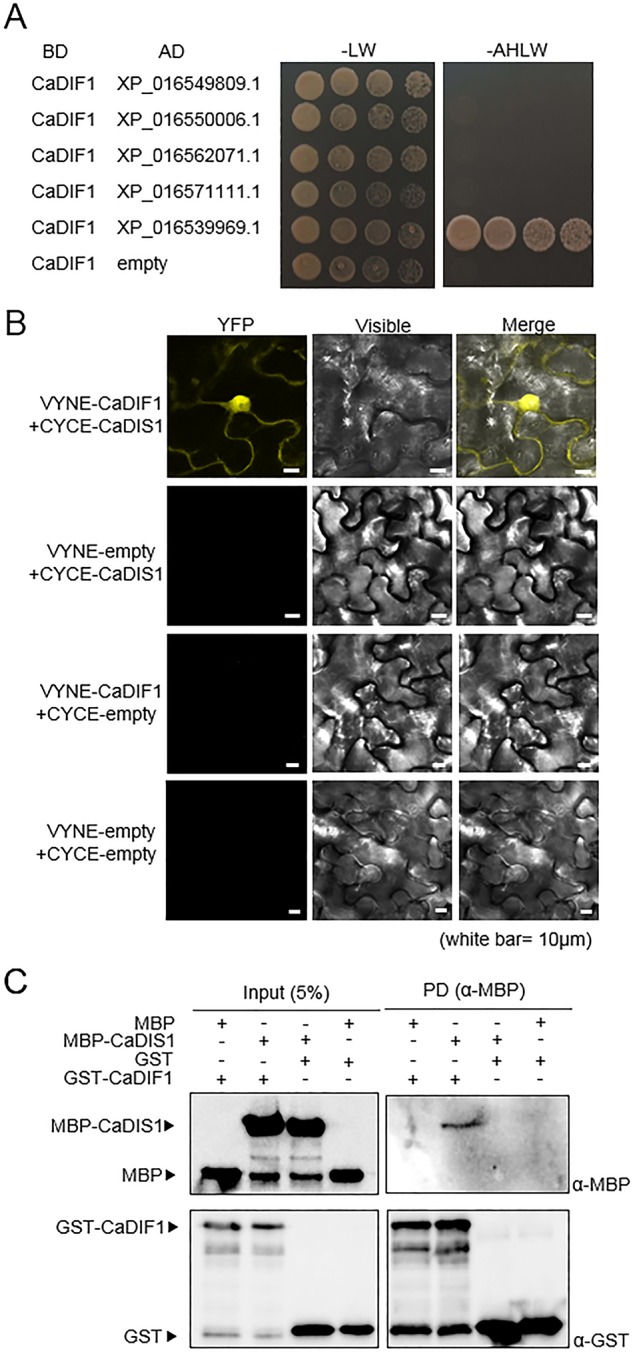
Interaction of CaDIF1 with CaDIS1. **(A)** Identification of the CaDIF1-interacting partner CaDIS1 (*C. annuum* DIF1-Interacting SKP 1). Yeast strain AH109 was transformed with pGBKT7-CaDIF1 as bait and pGADT7-CaSKPs as prey. Interaction was indicated by growth on selective medium (SC-Ade-His-Leu-Trp; right); growth on SC-Leu-Trp was used as a control (left). **(B)** Bimolecular fluorescence complementation (BiFC) assay of interactions between CaDIF1 and CaDIS1. CaDIF1-VYNE was co-expressed with CaDIS1-CYCE in the leaves of *N. benthamiana*. White bar = 10 μm. **(C)** Pull-down assay of interaction between CaDIF1 and CaDIS1.

To determine whether CaDIF1 is a component of SCF1 type E3 ligase complex, we performed Y2H assay using five pepper SKP proteins as prey and CaDIF1 as bait (**Figure 7A**). Yeast cell transformed with CaDIF1 and XP_016539969.1 grew on selective media, indicating that XP_016539969.1 interacts with CaDIF1 in yeast cells. We performed further analysis by designating XP_016539969.1 as CaDIS1 (*Capsicum annuum* DIF1-Interacting SKP 1). To confirm the interaction and localization of CaDIF1 and CaDIS1 *in vivo*, a bimolecular fluorescence complementation (BiFC) assay was performed (**Figure 7B**). The yellow fluorescence derived from co-expression of *CaDIF1*:VYNE with *CaDIS1*:CYCE localized constitutive strong nuclear and cytosolic localization. Consistent with these data, an *in vitro* pull-down assay verified that CaDIF1 interacts directly with CaDIS1 (**Figure 7C**). Taken together, these data demonstrate that CaDIF1 is able to interact with CaDIS1.

The CaDIS1 protein consists of 156 amino acids and have a conserved SKP1-like protein domain (including 4–106 amino acid residues) (**Supplementary Figure S2A**). Protein sequence assays showed that CaDIS1 has high homology with SKP1 proteins from other plants (**Supplementary Figures S2A, B**). Because the CaDIS1 is able to interact with CaDIF1, CaDIS1 plays a role as a positive regulator in ABA and drought signaling, we determined whether the transcripts accumulation of *CaDIS1* is induced by ABA or abiotic stress treatments. Drought treatment induced *CaDIS1* expression; however, unlike the expression patterns of *CaDIF1*, the *CaDIS1* transcripts did not accumulate more strongly after treatment with ABA, H_2_O_2_, or high salinity (**Supplementary Figure S3A**). When the fusion proteins of CaDIS1-GFP was expressed in the *N. benthamiana* epidermal cells, this protein was also localized to the nucleus, and cytoplasm (**Supplementary Figure S3B**).

### Reduced Drought Tolerance of *CaDIS1*-Silenced Pepper Plants

**Figure 8:**
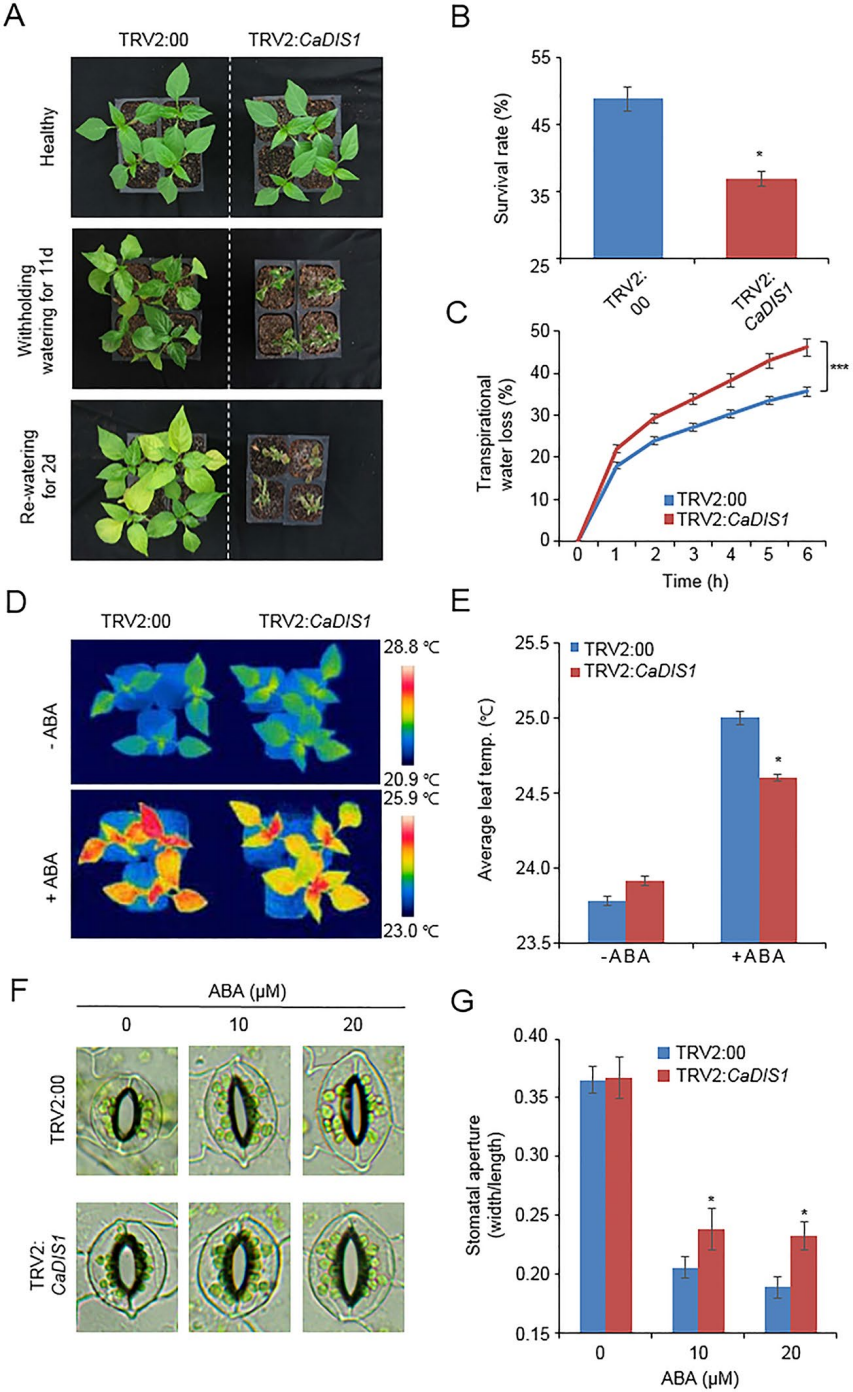
Reduced tolerance of *CaDIS1*-silenced pepper plants to drought stress. **(A)** Drought susceptibility of *CaDIS1*-silenced pepper plants. Four-week-old TRV2:*CaDIS1* and TRV2:00 pepper plants were subjected to drought stress by withholding watering for 11 days. Representative images were taken before (left) and after (middle) drought stress and 2 days after re-watering (right). **(B)** The survival rate was measured by counting the number of plants with green and rehydrated leaves 2 days after re-watering. Data represent the mean ± standard deviation of three independent biological replicates. **(C)** Transpirational water loss from the leaves of empty vector control and *CaDIS1*-silenced pepper plants at various times after detachment of leaves. Data represent the mean ± standard deviation of three independent biological replicates. **(D** and **E)** Leaf temperatures of empty vector control and *CaDIS1*-silenced pepper plants after treatment with 100 μM ABA. Representative thermographic images were taken 6 h after treatment **(D)** and the mean leaf temperature was measured using 10 plants of each line **(E)**. Data represent the mean ± SD of three independent biological replicates. **(F** and **G)** Stomatal apertures in empty vector control and *CaDIS1*-silenced pepper plants after ABA treatment. Representative images were taken under a microscope **(F)** and the stomatal apertures were measured **(G)** Leaf peels were harvested from 2-week-old pepper plants and incubated in stomatal opening solution containing 10 or 20 μM ABA; the stomatal apertures were then measured under a microscope. Representative images were taken before (left) and 2 h after (right) ABA treatment. Data represent the mean ± standard error of three independent biological replicates, each evaluating 20 plants. Asterisks indicate significant differences between the control and the *CaDIS1*-silenced pepper plants (Student’s *t*-test; *P* 0.05).

*CaDIF1*-knock down pepper plants displayed a sensitive phenotype to drought stress (**Figure 3**); therefore, we also postulated that *CaDIS1* is involved in the defence signaling to drought stress. To confirm this prediction, we conducted phenotypic analysis by VIGS assay in pepper plants (**Figure 8**). We examined *CaDIS1* knock down by RT-PCR assay of control (TRV2:00) and *CaDIS1*-knock down pepper (TRV2:*CaDIS1*) plants. The *CaDIS1* transcripts were lowly accumulated in the *CaDIS1*-knock down pepper leaves compared with the control leaves (**Supplementary Figure S1C**). To subject drought treatment to control and *CaDIS1*-knock down pepper plants, we withheld water for 11 days and re-watering for 2 days (**Figure 8A**). In comparison with control plants, *CaDIS1*-knock down pepper plants showed a wilted phenotype after drought stress and showed reduced drought tolerance after re-watering. The survival ratio of *CaDIS1*-knock down pepper plants was 36.9%, while the survival ratio of control pepper was approximately 48.7% (**Figure 8B**). Next, the transpirational water loss was compared by estimating the leaf fresh weights detached from control and *CaDIS1*-knock down pepper plants (**Figure 8C**). As same with the drought-sensitive phenotype, *CaDIS1*-knock down pepper leaves exhibited dramatically lower fresh weight than control leaves. Next, we measured leaf temperatures and stomatal apertures in the presence and absence of ABA (**Figures 8D, G**). In the absence of ABA, we were not able to detect different leaf temperatures significantly between control pepper and *CaDIS1*-knock down pepper plants (**Figures 8D, E**). However, after treatment with 100 µM ABA, the leaf temperatures of *CaDIS1*-silenced pepper were significantly lower than those of control plants. Consistent with leaf temperatures, the stomatal apertures were significantly larger in *CaDIS1*-silenced pepper than in control plants. (**Figures 8F, G**). These data demonstrate that the guard cells constituting stomatal pore of *CaDIS1*-knock down pepper plants display ABA hyposensitivity, and altered ABA sensitivity probably lead to reduced water retention in drought stress conditions.

## Discussion

A large number of studies have suggested that regulation of protein stability using the ubiquitin–proteasome system is crucial for transfer of ABA signaling and the drought stress response at multiple steps. Several E3 ligases, which are related to ABA signaling and the drought stress response, have been isolated (Li et al., 2016; Yu et al., 2016; Lim et al., 2017). Recent studies have reported that the stability of core ABA-signaling components—such as ABA receptors and group A PP2Cs—is modulated by different types of E3 ligases. The RING type E3 ligase RSL1 and the SCF type E3 ligase complexes RIFP1 and CRL4 interact with and ubiquitinate ABA receptors such as RCAR1, RCAR3, RCAR10, and RCAR11 (Bueso et al., 2014; Irigoyen et al., 2014; Li et al., 2016). PUB12 and PUB 13, which are U-box type E3 ligases, are involved in ubiquitination and degradation of ABI1 (Kong et al., 2015). RING type E3 ligases CaAIRF1, RGLG1, and RGLG5 are associated with stability of group A PP2Cs, including CaADIP1, CaAIPP1 PP2CA, HAB2, and ABI2 (Wu et al., 2016; Baek et al., 2017; Lim et al., 2017). In this study, we have determined that the pepper F-box protein CaDIF1—which is one of component of the SKP type E3 ligase complex—interacts with CaDIS1 and positively modulates ABA signaling and drought stress response.

Pepper plants have very low transformation efficiency; therefore, to determine the biological role of *CaDIF1* and *CaDIS1* in ABA signaling and response to drought stress, we subjected *CaDIF1*- and *CaDIS1*-silenced pepper plants, and *CaDIF1*-OX *Arabidopsis* plants to ABA treatment and drought stress. Under drought stress conditions, plants displayed adaptive processes, including ABA biosynthesis, which increases water retention and enhances drought tolerance by regulating transpirational water loss *via* stomatal opening/closure. In this condition, *CaDIF1*- and *CaDIS1*-silenced pepper plants exhibited more wilted phenotypes, and these were characterized by decreased leaf fresh weight and reduced stomatal closure (**Figures 3** and **8**). Conversely, *CaDIF1*-OX plants exhibited less wilted phenotypes, and these were characterized by increased water retention and enhanced stomatal closure (**Figure 5**). These data indicate that regulation of stomatal apertures in *CaDIF1*- and *CaDIS1*-knock down pepper plants, and in *CaDIF1*-OX transgenic plants confers reduced and enhanced drought-tolerant phenotypes, respectively.

Previous studies have shown that F-box proteins are involved in a large number of cellular processes in higher plants, such as tissue development and different hormone responses (Lechner et al., 2006; Fonseca et al., 2009; Schwechheimer and Willige, 2009). SCF type E3 ubiquitin ligase complex is composed of four subunits (Lechner et al., 2006). Among these, SKP1 protein interacts with CULLIN1 and F-box proteins. Most F-box proteins contain F-box domains in the N-terminus, and these domains have high variability in their amino acid composition; therefore, to determine the cellular function of these proteins, interaction assays are necessary (Deshaies, 1999; Koops et al., 2011). The CaDIF1 protein localized predominantly in the nucleus and CaDIS1-GFP fusion protein signal also detected in the nucleus. Moreover, our BiFC assay revealed that the interaction signals of both proteins were also observed in the nucleus. These results imply that CaDIF1 and CaDIS1 function as components of the SCF type E3 ubiquitin ligase complex. *CaDIF1*- and *CaDIS1*-silenced pepper plants exhibited hyposensitivity and reduced drought tolerance, whereas *CaDIF1*-overexpressing *Arabidopsis* plants displayed contrasting phenotypes. These data indicate that CaDIF1 and CaDIS1 function as positive modulators of ABA signaling and the response to drought stress. Moreover, the target proteins of SCF^CaDIF1^ ubiquitin ligase may negatively regulate ABA signaling and the drought stress response.

A large number of positive modulators of ABA induce mechanisms related to defence processes—including stress-related gene expression—thereby conducting adaptation in environmental stress condition (Yamaguchi-Shinozaki and Shinozaki, 2006; Cutler et al., 2010). In the present study, we were unable to identify the target protein of SCF ubiquitin ligase, which is composed of CaDIF1 and CaDIS1. Expression levels of stress-related genes indicated that CaDIF1 may regulate expression of *DREB2A*, *RAB18*, *RD20*, *RD29A*, and *RD29B* directly or indirectly, and that this protein likely acts modulator of these genes in the response to drought stress. However, the accumulation of *NCED3* transcripts did not differ significantly between wild-type and *CaDIF1*-OX plants grown under well-watered and drought stress conditions. Under stress conditions, *NCED3* functions in ABA biosynthesis, which is essential for the plant defence response to stress (Nambara and Marion-Poll, 2005; Urano et al., 2009). Several studies have shown that *NCED3* gene expression induces stress-related gene expression and increases endogenous ABA levels (Iuchi et al., 2001; Lim et al., 2017). These results suggest that phenotypic variations may not be caused by differences in ABA levels but may be derived from changes in the ABA-signaling process. Moreover, enhanced expression *CaDIF1* did not influence the induction level of group A PP2Cs (**Figure 6**), which function as negative regulators of ABA signaling. Several studies have elucidated that the ABA-signal transduction pathway—from ABA perception to defence response to environmental stress (Lee and Luan, 2012). ABA perception by ABA receptors (PYR/PYLs/RCARs) and downstream ABA-signaling modulators—including group A PP2Cs and SnRK2 type kinases—are conserved in higher plants (Klingler et al., 2010; Umezawa et al., 2010; Kim et al., 2012); hence ABA receptors and other ABA-signaling components presumably play critical roles in adaptation to stress (Gonzalez-Guzman et al., 2012; Lee and Luan, 2012; Lim et al., 2013; Ding et al., 2015). On the basis of these results, it might be speculated that altered expression of *CaDIF1* is unable to influence ABA biosynthesis and events occurring upstream of ABA signaling, but does influence events related to stress-related gene expression—located downstream of PP2Cs—leading to changes in ABA sensitivity and drought tolerance. Since CaDIF1 and CaDIS1 could function as components of the SCF type E3 ubiquitin ligase complex, we also did not rule out the possibility of the CaDIF1 and CaDIS1-mediated regulation in the protein stability of NCED3, PP2Cs or upstream transcriptional regulators of the stress-related genes.

In conclusion, our studies suggest that CaDIF1 and CaDIS1 positively regulate in the drought stress response through changes in ABA sensitivity, including induction of stomatal closure and stress-related gene expression. We have elucidated the biological functions of CaDIF1 and CaDIS1 in the drought stress response; however, the precise mechanisms whereby these proteins function remain unclear. Therefore, to clarify the functions of CaDIF1 and CaDIS1 under drought stress conditions, further studies to identify the direct target proteins are required.

## Materials and Methods

### Plant Materials and Growth Conditions

Seeds of pepper (*C. annuum* L., “Nockwang”), tobacco (*N. benthamiana*), and *A. thaliana* (ecotype Col-0) were sown in a steam-sterilized compost soil mix (peat moss, perlite, and vermiculite, 5:3:2, v/v/v), sand, and loam soil (1:1:1, v/v/v). The plants were raised at 25 ± 1°C under a 16 h light/8 h dark photoperiod (130 μmol photons·m^−2^·s^−1^). *Arabidopsis* [wild-type (ecotype Col-0) and *CaDIF1*-OX] seeds were grown on MS medium containing 1% sucrose. Details of plasmid construction and transgenic plants were described below.

### Virus-Induced Gene Silencing (VIGS)

VIGS analysis was performed to knockdown *CaDIF1* and *CaDIS1* in *C. annuum* (pepper) as described previously (Lim et al., 2017). A 254 bp (1–254 nucleotide sequences) fragment of the *CaDIF1* or a 299 bp (2–300 nucleotide sequences) fragment of the *CaDIS1* cDNA was inserted into the pTRV2 vector and introduced into *Agrobacterium tumefaciens* strain GV3101 *via* electroporation. *A. tumefaciens* strain GV3101 containing pTRV1, pTRV2:00, pTRV2:*CaDIF1*, or pTRV2:*CaDIS1* was infiltrated to the fully expanded pepper cotyledons (OD_600_ = 0.2, each from different construct).

### Generation of *CaDIF1*-OX Plants

*Agrobacterium* transformation into the *A. thaliana* was conducted by using the floral dip method (Clough and Bent, 1998); all mutants generated in this study were in the Col-0 background. For *CaDIF1*-OX, full-length cDNA sequences were integrated into the pBIN35SGFP binary vector. For selection of overexpressing plants, we used 25 μg·ml^−1^ phosphinothricin.

### ABA, Drought, NaCl, and H_2_O_2_ Treatments

To analyze *CaDIF1* and *CaDIS1* expression in pepper plants, we treated with ABA (100 μM), H_2_O_2_ (100 μM), drought, or NaCl (200 mM) as described previously (Lim et al., 2015a; Lim et al., 2015b). Pepper leaves were harvested at 0, 2, 6, 12, and 24 h after each treatment. For the determination of ABA sensitivity, we measured the seed germination rates, cotyledon development, and primary root lengths on ABA containing MS plants. For drought sensitivity analysis, we used 4-week-old pepper and 3-week-old *Arabidopsis* plants and subjected to drought stress by withholding water for 11 and 14 days, respectively. After re-watering for 2 days and 1 day, respectively, we calculated the survival. To determine drought sensitivity or tolerance, transpirational water loss was measured. Pepper and *Arabidopsis* rosette leaves were detached, and the reduced fresh weight was measured for each time points. For quantitative RT-PCR assay, 4-week-old *CaDIF1*-OX transgenic *Arabidopsis* plants were subjected to drought stress. All the experiments were conducted three times.

### Measurements of Stomatal Apertures and Thermal Imaging

Leaf temperatures and stomatal apertures were measured as described previously (Lim and Lee, 2016). Briefly, *A. thaliana* and pepper leaf peels were floated in buffer with 10 μM CaCl_2_, 10 mM MES–KOH (pH 6.15), and 50 mM KCl, in day condition. After incubation for 3 h, stomata begins to close by replacing the buffer with 10 and 20 μM of ABA. Then after 2.5, 100 stomata apertures per 10 leaves were measured under a Nikon Eclipse 80i microscope. To analyze the thermal imaging, we used full expanded first and second leaves of pepper plants and *Arabidopsis* were treated with 100 μM ABA. For measurement of leaf temperatures, we used infrared camera (T420; FLIR) equipped with FLIR ver 5.2 software. All the experiments were conducted three times.

### Transient CaDIF1-GFP and CaDIS1-GFP Fusion Proteins Expression in *N. Benthamiana*

To investigate subcellular localization and BiFC assay of CaDIF1 and CaDIS1, we used the open reading frame of *CaDIF1*, *CaDIS1*, and pBIN35SGFP, Pro-35S:VYNE, and Pro-35S:CYCE vectors, which express the proteins driven by the *35S* promoter. The different constructs were introduced into the *A. tumefaciens* strain GV3101 and selected by 50 μg·ml^−1^ kanamycin. These cells were combined with the silencing suppressor p19 and introduced into the *N. benthamiana* epidermal cells by using agroinfiltration method. At 3 days after infiltration, leaf discs were cut, and the lower epidermal cells were examined under a confocal microscope (510 UV/Vis Meta; Zeiss) equipped with LSM Image Browser software.

### Yeast-Two Hybrid Assay

Yeast-two hybrid (Y2H) assays were performed as described previously (Lim et al., 2017). The cDNA fragments of *CaDIF1 or CaDIS1* were subcloned into the pGBKT7 or pGADT7 vectors, respectively, and co-transformed into yeast strain AH109 using the lithium acetate-mediated transformation method (Ito et al., 1983). To evaluate the interaction between bait and prey proteins, transformant candidates were selected on SC-Leu-Trp medium. Next, 10-fold serial dilutions were prepared from each yeast cell culture (OD_600_ = 0.5), and 5 μl of each sample was spotted onto SC-Leu-Trp medium or SC-Ade-His-Leu-Trp medium. Yeast colonies were incubated at 30°C for 5 days.

### Expression of the MBP-CaDIS1 and GST‐CaDIF1 Recombinant Proteins in Bacterial Cells and Pull‐Down Assay

For expression of the maltose‐binding protein (MBP) and glutathione‐S‐transferase (GST) recombinant proteins, the pMAL‐c2X (New England Biolabs) and pGEX‐4T‐3 (GE Healthcare) vectors were respectively used. Each vector, harboring the full‐length cDNA sequence of *CaDIS* or *CaDIF1*, was introduced into *Escherichia coli* strain BL21 cells. The fusion proteins were induced and purified according to the manufacturer’s instructions.

For the pull‐down assay, 5 μg of GST or GST‐CaDIF1 were incubated with 5 μg of MBP or MBP‐CaDIS1 for 2 h at 4°C on constant rocking in 0.5 ml of binding buffer (20 mM Tris–HCl, pH 7.5, 200 mM NaCl, 1 mM EDTA, 0.5% Tween‐20) containing GST resin. After boiling at 97°C for 5 min, eluted proteins were analyzed using SDS‐PAGE, followed by western blotting and immunodetection with anti‐MBP and anti‐GST antibodies.

## Data Availability Statement

All datasets generated for this study are included in the article/**Supplementary Material**.

## Author Contributions

JL and CWL performed the experiments and analyzed the results. SCL designed the experiments and wrote the manuscript.

## Conflict of Interest

The authors declare that the research was conducted in the absence of any commercial or financial relationships that could be construed as a potential conflict of interest.

## Supplementary Material

Figure S1 Expression level of CaDIF1 in overexpressing Arabidopsis transgenic plants. RT-PCR analysis of CaDIF1 expression in wild-type plants and CaDIF1-OX transgenic lines. Actin8 was used as an internal control gene.

Figure S2 Homology of the pepper CaDIS1 (Capsicum annuum DIF Interacting SKP 1) protein. (A) Alignment of the deduced amino acid sequences of CaDIS1 with its homologous proteins was performed using ClustalW2. Identical and similar amino acid residues are shaded according to the percentage identity in ClustalW2. Sequences include CaDIS1 (accession no. XP_016539969.1), Arachis duranensis (accession no. XP_020996771.1), Solanum lycopersicum (accession no. XP_004250723.1), Nicotiana tomentosiformis (accession no. XP_009620406.1.1), and Arabidopsis thaliana (accession no. NP_568603.1). The box indicates the SKP1 like protein domain (B) Phylogenetic tree analysis of CaDIS1protein. BLAST search was performed by using deduced amino acid sequences of CaDIS1, and sequences with highest similarity were gathered from each plant species. Multiple sequence alignment was performed using Clustal Omega (http://www.ebi.ac.uk/Tools/msa/clustalo/) with the default settings. The phylogenetic tree was drawn using the neighbor-joining method with MEGA software (version 7.0). Bootstrap values were calculated from 1000 bootstrap replications and were indicated at each branch point. Scale bar indicates genetic distance.

Figure S3 Expression of the CaDIS1 gene and localization of the CaDIS1 protein. (A) Induction of CaDIS1 in pepper leaves at various time points after treatment with drought, 100 μM abscisic acid (ABA), H2O2 (100 μm) and NaCl (200 mm). The pepper Actin1 genes were used as internal control. (B) Subcellular localization of the CaDIS1 protein using transient expression of the green fluorescent protein (GFP) fusion protein in Nicotiana benthamiana cells. The 35S: CaDIS1-GFP construct was expressed using agroinfiltration of N. benthamiana leaves and observed under a confocal laser-scanning microscope. 4´,6-Diamidino-2-phenylindole (DAPI) staining was used as a marker for the nucleus. White bar = 10 μm.

## References

[B1] Baek W. Lim C. W. Lee S. C. (2017). Functional analysis of the pepper protein phosphatase, CaAIPP1, and its interacting partner CaAIRF1: Modulation of ABA signalling and the drought stress response. Plant Cell Environ. 40 (10), 2359–2368. 10.1111/pce.13039 28742940

[B2] Bueso E. Rodriguez L. Lorenzo-Orts L. Gonzalez-Guzman M. Sayas E. Munoz-Bertomeu J. (2014). The single-subunit RING-type E3 ubiquitin ligase RSL1 targets PYL4 and PYR1 ABA receptors in plasma membrane to modulate abscisic acid signaling. Plant J. 80 (6), 1057–1071. 10.1111/tpj.12708 25330042

[B3] Chen Y. Chi Y. Meng Q. Wang X. Yu D. (2018). GmSK1, an SKP1 homologue in soybean, is involved in the tolerance to salt and drought. Plant Physiol. Biochem. 127, 25–31. 10.1016/j.plaphy.2018.03.007 29544210

[B4] Cheong Y. H. Pandey G. K. Grant J. J. Batistic O. Li L. Kim B. G. (2007). Two calcineurin B-like calcium sensors, interacting with protein kinase CIPK23, regulate leaf transpiration and root potassium uptake in Arabidopsis. Plant J. 52 (2), 223–239. TPJ3236 [pii] 17922773 10.1111/j.1365-313X.2007.03236.x

[B5] Ciechanover A. Schwartz A. L. (1998). The ubiquitin-proteasome pathway: the complexity and myriad functions of proteins death. Proc. Natl. Acad. Sci. U. S. A. 95 (6), 2727–2730. 10.1073/pnas.95.6.2727 9501156 PMC34259

[B6] Clough S. J. Bent A. F. (1998). Floral dip: a simplified method for Agrobacterium-mediated transformation of *Arabidopsis thaliana* . Plant J. 16 (6), 735–743. 10.1046/j.1365-313x.1998.00343.x 10069079

[B7] Cutler S. R. Rodriguez P. L. Finkelstein R. R. Abrams S. R. (2010). Abscisic acid: emergence of a core signaling network. Annu. Rev. Plant Biol. 61, 651–679. 10.1146/annurev-arplant-042809-112122 20192755

[B8] Deshaies R. J. (1999). SCF and Cullin/Ring H2-based ubiquitin ligases. Annu. Rev. Cell Dev. Biol. 15, 435–467. 10.1146/annurev.cellbio.15.1.435 10611969

[B9] Ding Y. Li H. Zhang X. Xie Q. Gong Z. Yang S. (2015). OST1 kinase modulates freezing tolerance by enhancing ICE1 stability in Arabidopsis. Dev. Cell 32 (3), 278–289. 10.1016/j.devcel.2014.12.023 25669882

[B10] Fonseca S. Chico J. M. Solano R. (2009). The jasmonate pathway: the ligand, the receptor and the core signalling module. Curr. Opin. Plant Biol. 12 (5), 539–547. 10.1016/j.pbi.2009.07.013 19716757

[B11] Gagne J. M. Downes B. P. Shiu S. H. Durski A. M. Vierstra R. D. (2002). The F-box subunit of the SCF E3 complex is encoded by a diverse superfamily of genes in Arabidopsis. Proc. Natl. Acad. Sci. U. S. A. 99 (17), 11519–11524. 10.1073/pnas.162339999 12169662 PMC123288

[B12] Goda H. Sasaki E. Akiyama K. Maruyama-Nakashita A. Nakabayashi K. Li W. (2008). The AtGenExpress hormone and chemical treatment data set: experimental design, data evaluation, model data analysis and data access. Plant J. 55 (3), 526–542. 10.1111/j.0960-7412.2008.03510.xTPJ3510 [pii] 18419781

[B13] Gonzalez-Guzman M. Pizzio G. A. Antoni R. Vera-Sirera F. Merilo E. Bassel G. W. (2012). Arabidopsis PYR/PYL/RCAR receptors play a major role in quantitative regulation of stomatal aperture and transcriptional response to abscisic acid. Plant Cell 24 (6), 2483–2496. 10.1105/tpc.112.098574 22739828 PMC3406898

[B14] Irigoyen M. L. Iniesto E. Rodriguez L. Puga M. I. Yanagawa Y. Pick E. (2014). Targeted degradation of abscisic acid receptors is mediated by the ubiquitin ligase substrate adaptor DDA1 in Arabidopsis. Plant Cell 26 (2), 712–728. 10.1105/tpc.113.122234 24563205 PMC3967035

[B15] Ito H. Fukuda Y. Murata K. Kimura A. (1983). Transformation of intact yeast cells treated with alkali cations. J. Bacteriol. 153 (1), 163–168. 6336730 10.1128/jb.153.1.163-168.1983 PMC217353

[B16] Iuchi S. Kobayashi M. Taji T. Naramoto M. Seki M. Kato T. (2001). Regulation of drought tolerance by gene manipulation of 9-cis-epoxycarotenoid dioxygenase, a key enzyme in abscisic acid biosynthesis in Arabidopsis. Plant J. 27 (4), 325–333. tpj1096 [pii] 11532178 10.1046/j.1365-313x.2001.01096.x

[B17] Jakab G. Ton J. Flors V. Zimmerli L. Métraux J. P. Mauch-Mani B. (2005) Enhancing Arabidopsis salt and drought stress tolerance by chemical priming for its abscisic acid responses. Plant physiol. 139 (1), 267–274. 10.1104/pp.105.065698 16113213 PMC1203376

[B18] Joo H. Lim C.W. Lee S. C. (2019) A pepper RING-type E3 ligase, CaASRF1, plays a positive role in drought tolerance via modulation of CaAIBZ1 stability. Plant J. 98 (1), 5–18. 10.1111/tpj.14191 30548716

[B19] Kim H. Hwang H. Hong J. W. Lee Y. N. Ahn I. P. Yoon I. S. (2012). A rice orthologue of the ABA receptor, OsPYL/RCAR5, is a positive regulator of the ABA signal transduction pathway in seed germination and early seedling growth. J. Exp. Bot. 63 (2), 1013–1024. 10.1093/jxb/err338err338[pii] 22071266

[B20] Kipreos E. T. Pagano M. (2000). The F-box protein family. Genome Biol. 1 (5), REVIEWS3002. 10.1186/gb-2000-1-5-reviews3002 11178263 PMC138887

[B21] Klingler J. P. Batelli G. Zhu J. K. (2010). ABA receptors: the START of a new paradigm in phytohormone signalling. J. Exp. Bot. 61 (12), 3199–3210. 10.1093/jxb/erq151erq151 [pii] 20522527 PMC3107536

[B22] Kong L. Cheng J. Zhu Y. Ding Y. Meng J. Chen Z. (2015). Degradation of the ABA co-receptor ABI1 by PUB12/13 U-box E3 ligases. Nat. Commun. 6, 8630. 10.1038/ncomms9630ncomms9630[pii] 26482222 PMC4667695

[B23] Koops P. Pelser S. Ignatz M. Klose C. Marrocco-Selden K. Kretsch T. (2011). EDL3 is an F-box protein involved in the regulation of abscisic acid signalling in Arabidopsis thaliana. J. Exp. Bot. 62 (15), 5547–5560. 10.1093/jxb/err236 21831845 PMC3223051

[B24] Kumar S. Stecher G. Tamura K. (2016). MEGA7: Molecular Evolutionary Genetics Analysis Version 7.0 for Bigger Datasets. Mol. Biol. Evol. 33 (7), 1870–1874. 10.1093/molbev/msw054 27004904 PMC8210823

[B25] Lechner E. Achard P. Vansiri A. Potuschak T. Genschik P. (2006). F-box proteins everywhere. Curr. Opin. Plant Biol. 9 (6), 631–638. 10.1016/j.pbi.2006.09.003 17005440

[B26] Lee S. C. Luan S. (2012). ABA signal transduction at the crossroad of biotic and abiotic stress responses. Plant Cell Environ. 35 (1), 53–60. 10.1111/j.1365-3040.2011.02426.x 21923759

[B27] Li Y. Zhang L. Li D. Liu Z. Wang J. Li X. (2016). The Arabidopsis F-box E3 ligase RIFP1 plays a negative role in abscisic acid signalling by facilitating ABA receptor RCAR3 degradation. Plant Cell Environ. 39 (3), 571–582. 10.1111/pce.12639 26386272

[B28] Lim C. W. Baek W. Han S. W. Lee S. C. (2013). Arabidopsis PYL8 plays an important role for ABA signaling and drought stress responses. Plant Pathol. J. 29 (4), 471–476. 10.5423/PPJ.NT.07.2013.0071 25288979 PMC4174817

[B29] Lim C. W. Baek W. Jung J. Kim J. H. Lee S. C. (2015a). Function of ABA in stomatal defense against biotic and drought stresses. Int. J. Mol. Sci. 16 (7), 15251–15270. 10.3390/ijms160715251ijms160715251[pii] 26154766 PMC4519898

[B30] Lim C. W. Baek W. Lee S. C. (2017). The Pepper RING-Type E3 Ligase CaAIRF1 regulates ABA and drought signaling *via* CaADIP1 protein phosphatase degradation. Plant Physiol. 173 (4), 2323–2339. 10.1104/pp.16.01817 28184010 PMC5373060

[B31] Lim C. W. Lee S. C. (2016). Pepper protein phosphatase type 2C, CaADIP1 and its interacting partner CaRLP1 antagonistically regulate ABA signalling and drought response. Plant Cell Environ. 39 (7), 1559–1575. 10.1111/pce.12721 26825039

[B32] Lim C. W. Lim S. Baek W. Han S. W. Lee S. C. (2015b). Expression and functional roles of the pepper pathogen-induced bZIP transcription factor, CabZIP2, in enhanced disease resistance to bacterial pathogen infection. Mol. Plant Microbe Interact. 28(7), 825–33. 10.1094/MPMI-10-14-0313-R 25738319

[B33] Mizuno T. Yamashino T. (2008). Comparative transcriptome of diurnally oscillating genes and hormone-responsive genes in Arabidopsis thaliana: insight into circadian clock-controlled daily responses to common ambient stresses in plants. Plant Cell Physiol. 49 (3), 481–487. 10.1093/pcp/pcn008pcn008[pii] 18202002

[B34] Nambara E. Marion-Poll A. (2005). Abscisic acid biosynthesis and catabolism. Annu. Rev. Plant Biol. 56, 165–185. 10.1146/annurev.arplant.56.032604.144046 15862093

[B35] Park S. Y. Peterson F. C. Mosquna A. Yao J. Volkman B. F. Cutler S. R. (2015). Agrochemical control of plant water use using engineered abscisic acid receptors. Nature 520 (7548), 545–548. 10.1038/nature14123nature14123 [pii] 25652827

[B36] Pazhouhandeh M. Molinier J. Berr A. Genschik P. (2011). MSI4/FVE interacts with CUL4-DDB1 and a PRC2-like complex to control epigenetic regulation of flowering time in Arabidopsis. Proc. Natl. Acad. Sci. U. S. A. 108 (8), 3430–3435. 10.1073/pnas.10182421081018242108 [pii] 21282611 PMC3044392

[B37] Sadanandom A. Bailey M. Ewan R. Lee J. Nelis S. (2012). The ubiquitin-proteasome system: central modifier of plant signalling. New Phytol. 196 (1), 13–28. 10.1111/j.1469-8137.2012.04266.x 22897362

[B38] Schwechheimer C. Willige B. C. (2009). Shedding light on gibberellic acid signalling. Curr. Opin. Plant Biol. 12 (1), 57–62. 10.1016/j.pbi.2008.09.004 18930434

[B39] Seo K. I. Lee J. H. Nezames C. D. Zhong S. Song E. Byun M. O. (2014). ABD1 is an Arabidopsis DCAF substrate receptor for CUL4-DDB1-based E3 ligases that acts as a negative regulator of abscisic acid signaling. Plant Cell 26 (2), 695–711. 10.1105/tpc.113.119974tpc.113.119974 [pii] 24563203 PMC3967034

[B40] Sirichandra C. Wasilewska A. Vlad F. Valon C. Leung J. (2009). The guard cell as a single-cell model towards understanding drought tolerance and abscisic acid action. J. Exp. Bot. 60 (5), 1439–1463. 10.1093/jxb/ern340ern340 [pii] 19181866

[B41] Stone S. L. Hauksdottir H. Troy A. Herschleb J. Kraft E. Callis J. (2005). Functional analysis of the RING-type ubiquitin ligase family of Arabidopsis. Plant Physiol. 137 (1), 13–30. 137/1/13 [pii]10.1104/pp.104.052423 15644464 10.1104/pp.104.052423 PMC548835

[B42] Umezawa T. Nakashima K. Miyakawa T. Kuromori T. Tanokura M. Shinozaki K. (2010). Molecular basis of the core regulatory network in ABA responses: sensing, signaling and transport. Plant Cell Physiol. 51 (11), 1821–1839. 10.1093/pcp/pcq156pcq156 [pii] 20980270 PMC2978318

[B43] Urano K. Maruyama K. Ogata Y. Morishita Y. Takeda M. Sakurai N. (2009). Characterization of the ABA-regulated global responses to dehydration in Arabidopsis by metabolomics. Plant J. 57 (6), 1065–1078. 10.1111/j.1365-313X.2008.03748.x 19036030

[B44] Vierstra R. D. (2009). The ubiquitin-26S proteasome system at the nexus of plant biology. Nat. Rev. Mol. Cell Biol. 10 (6), 385–397. 10.1038/nrm2688nrm2688 [pii] 19424292

[B45] Wu Q. Zhang X. Peirats-Llobet M. Belda-Palazon B. Wang X. Cui S. (2016). Ubiquitin Ligases RGLG1 and RGLG5 Regulate Abscisic Acid Signaling by Controlling the Turnover of Phosphatase PP2CA. Plant Cell 28 (9), 2178–2196. 10.1105/tpc.16.00364 27577789 PMC5059804

[B46] Yamaguchi-Shinozaki K. Shinozaki K. (2006). Transcriptional regulatory networks in cellular responses and tolerance to dehydration and cold stresses. Annu. Rev. Plant Biol. 57, 781–803. 10.1146/annurev.arplant.57.032905.105444 16669782

[B47] Yu F. Wu Y. Xie Q. (2016). Ubiquitin-p-roteasome system in ABA signaling: from perception to action. Mol. Plant 9 (1), 21–33. 10.1016/j.molp.2015.09.015 26455462

[B48] Zhang X. Zhang L. Dong F. Gao J. Galbraith D. W. Song C. P. (2001). Hydrogen peroxide is involved in abscisic acid-induced stomatal closure in *Vicia faba*. Plant Physiol. 126 (4), 1438–1448. 10.1104/pp.126.4.1438 11500543 PMC117144

