# Supplementary figures and images for: Functional Analysis of Pepper F-box Protein CaDIF1 and Its Interacting Partner CaDIS1: Modulation of ABA Signaling and Drought Stress Response
Source: Front Plant Sci. 2019 Oct 30;10:1365. doi: 10.3389/fpls.2019.01365 (PMC6831560; doi:10.3389/fpls.2019.01365)

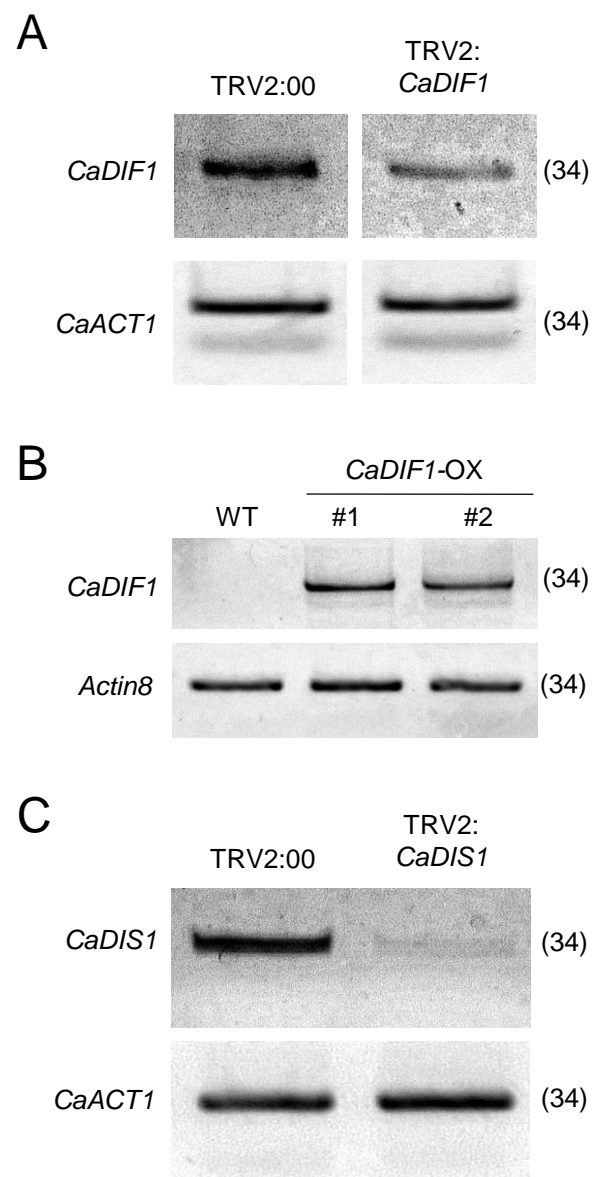

Fig. S1 Lim et al.

Supplement: Figure S1 — Expression level of CaDIF1 in overexpressing Arabidopsis transgenic plants. RT-PCR analysis of CaDIF1 expression in wild-type plants and CaDIF1-OX transgenic lines. Actin8 was used as an internal control gene. [file Image_1.pdf]

A

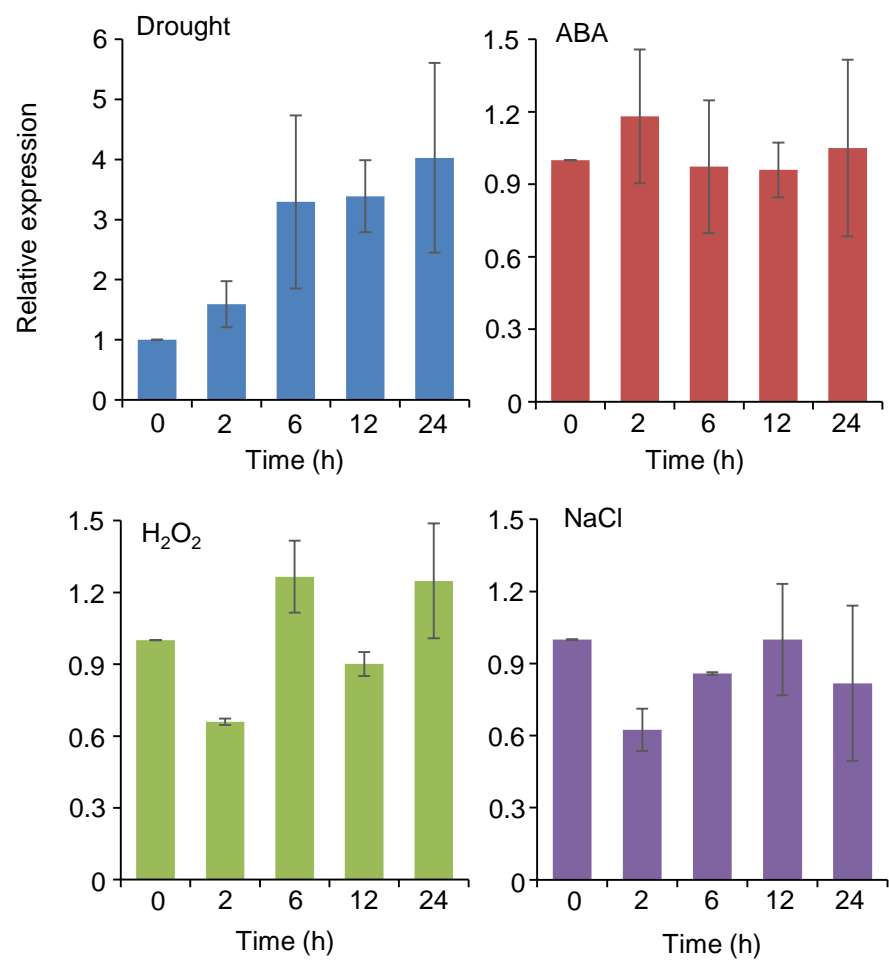

B

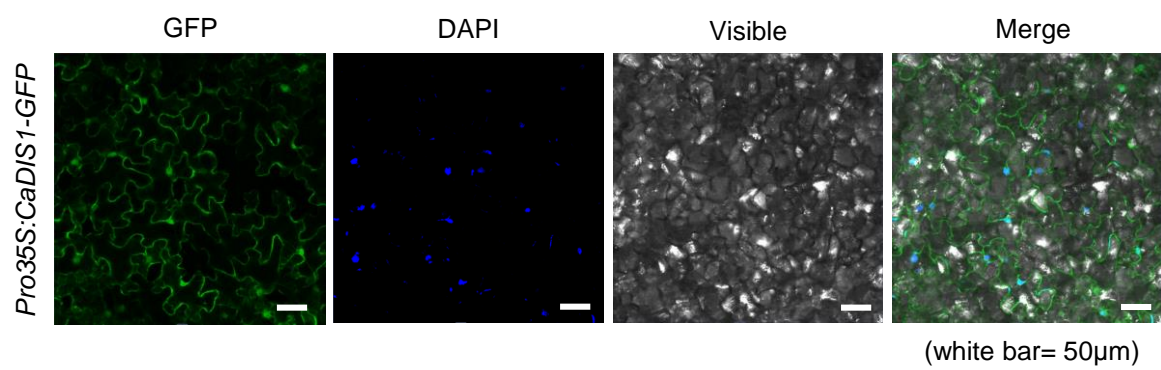

Fig. S3 Lim et al.

Supplement: Figure S3 — Expression of the CaDIS1 gene and localization of the CaDIS1 protein. (A) Induction of CaDIS1 in pepper leaves at various time points after treatment with drought, 100 μM abscisic acid (ABA), H2O2 (100 μm) and NaCl (200 mm). The pepper Actin1 genes were used as internal control. (B) Subcellular localization of the CaDIS1 protein using transient expression of the green fluorescent protein (GFP) fusion protein in Nicotiana benthamiana cells. The 35S: CaDIS1-GFP construct was expressed using agroinfiltration of N. benthamiana leaves and observed under a confocal laser-scanning microscope. 4´,6-Diamidino-2-phenylindole (DAPI) staining was used as a marker for the nucleus. White bar = 10 μm. [file Image_3.pdf]
