# Supplementary material for: Functional Analysis of Pepper F-box Protein CaDIF1 and Its Interacting Partner CaDIS1: Modulation of ABA Signaling and Drought Stress Response
Source: Front Plant Sci. 2019 Oct 30;10:1365. doi: 10.3389/fpls.2019.01365 (PMC6831560; doi:10.3389/fpls.2019.01365)
Supplement: Supplementary file 4 [file Table_1.pdf]

Supplemental Table S1. Sequences of primers used in this study

Primer name	Primer sequence (5'-3')
For cloning	
CaDIF1	Forward: ATGAGTGAGGTCCCGAGTCAAAGAC
(CA10g14050)	Reverse: CTATGTGGGCTCAACGATCTGATTT
(w/o stop codon)	Forward: GGTCGAATTCGCCCTTTGTGGGCTCAACGATC
	Reverse: GATCGTTGAGCCCACAAAGGGCGAATTCGACC
CaDIS1	Forward: ATGTCTGCCCCAAAGAAAATCT
(CA08g19370)	Reverse: TCACTCAAAGGCCCAAGCATT
(w/o stop codon)	Reverse: CTCAAAGGCCCAAGCATTC
For quantitative RT-PCR	
CaDIF1	Forward: ATGAGTGAGGTCCCGAGTCAAAGAC
	Reverse: CGGAGCCTTCGATTAACCGAT
CaDIS1	Forward: ATGTCTGCCCCAAAGAAAATCT
	Reverse: CCGCATCAACATGCTTCTTA
CaACT1	Forward: GACGTGACCTAACTGATAACCTGAT
(CA12g08730)	Reverse: CTCTCAGCACCAATGGTAATAACTT
AtActin8	Forward: CAACTATGTTCTCAGGTATTGCAGA
(At1g49240)	Reverse: GTCATGGAAACGATGTCTCTTTAGT
NCED3	Forward: ACATGGAAATCGGAGTTACAGATAG
(At3g14440)	Reverse: AGAAACAACAAACAAGAAACAGAGC
DREB2A	Forward: CTACAAAGCCTCAACTACGGAATAC
(At5g05410)	Reverse: AAACTCGGATAGAGAATCAACAGTC
RAB18	Forward: GGAAGAAGGGAATAACACAAAAGAT
(At5g66400)	Reverse: GCGTTACAAACCCTCATTATTTTTA
RD20	Forward: TGGTTTCCTATCTAAAGAAGCTGTG
(At2g33380)	Reverse: ATACAAATCCCCAAACTGAATAACA
RD29A	Forward: CACAATCACTTGGCTCCACTGTTG
(At5g52310)	Reverse: ACCTAGTAGCTGGTATGGAGGAACT
RD29B	Forward: GTTGAAGAGTCTCCACAATCACTTG
(At5g52300)	Reverse: ATACAAATCCCCAAACTGAATAACA
ABI1	Forward: GTTTGGGATGTAATGACGGATG
(At4g26080)	Reverse: TGAACTGAGGCAGAGAGGGTCC
ABI2	Forward: AGAAAAGAGGAGAAGGAAAAGATCC
(At5g57050)	Reverse: TAAAGAGAATTTTTACCCACCATCA
HAB1	Forward: GACTACCTCTCAATGCTTGCTCTAC
(At1g72770)	Reverse: AAAAACCTGTCGAAATTAGATCCTT
For semi-quantitative RT-PCR	
CaDIF1	Forward: ATGAGTGAGGTCCCGAGTCAAAGAC
	Reverse: CTATGTGGGCTCAACGATCTGATTT
CaDIS1	Forward: TAGAGGCCCCTTCCCTCAG
	Reverse: TAGGATTTAAACAAATCTTCTCATTG
For VIGS	
XbaI-CaDIF1	Forward: TCTAGAATGAGTGAGGTCCCGAGTCA
XhoI-CaDIF1	Reverse: CTCGAGAACGATGCTATCATGCGT
XbaI-CaDIS1	Forward: TCTAGATGTCTGCCCCAAAGAAAATC
XhoI-CaDIS1	Reverse: CTCGAGAGCCAGAATGAGATCGAAGA
