# Supplementary material for: Functional Analysis of Pepper F-box Protein CaDIF1 and Its Interacting Partner CaDIS1: Modulation of ABA Signaling and Drought Stress Response
Source: Front Plant Sci. 2019 Oct 30;10:1365. doi: 10.3389/fpls.2019.01365 (PMC6831560; doi:10.3389/fpls.2019.01365)
Supplement: Figure S2 — Homology of the pepper CaDIS1 (Capsicum annuum DIF Interacting SKP 1) protein. (A) Alignment of the deduced amino acid sequences of CaDIS1 with its homologous proteins was performed using ClustalW2. Identical and similar amino acid residues are shaded according to the percentage identity in ClustalW2. Sequences include CaDIS1 (accession no. XP_016539969.1), Arachis duranensis (accession no. XP_020996771.1), Solanum lycopersicum (accession no. XP_004250723.1), Nicotiana tomentosiformis (accession no. XP_009620406.1.1), and Arabidopsis thaliana (accession no. NP_568603.1). The box indicates the SKP1 like protein domain (B) Phylogenetic tree analysis of CaDIS1protein. BLAST search was performed by using deduced amino acid sequences of CaDIS1, and sequences with highest similarity were gathered from each plant species. Multiple sequence alignment was performed using Clustal Omega (http://www.ebi.ac.uk/Tools/msa/clustalo/) with the default settings. The phylogenetic tree was drawn using the neighbor-joining method with MEGA software (version 7.0). Bootstrap values were calculated from 1000 bootstrap replications and were indicated at each branch point. Scale bar indicates genetic distance. [file Image_2.pdf]

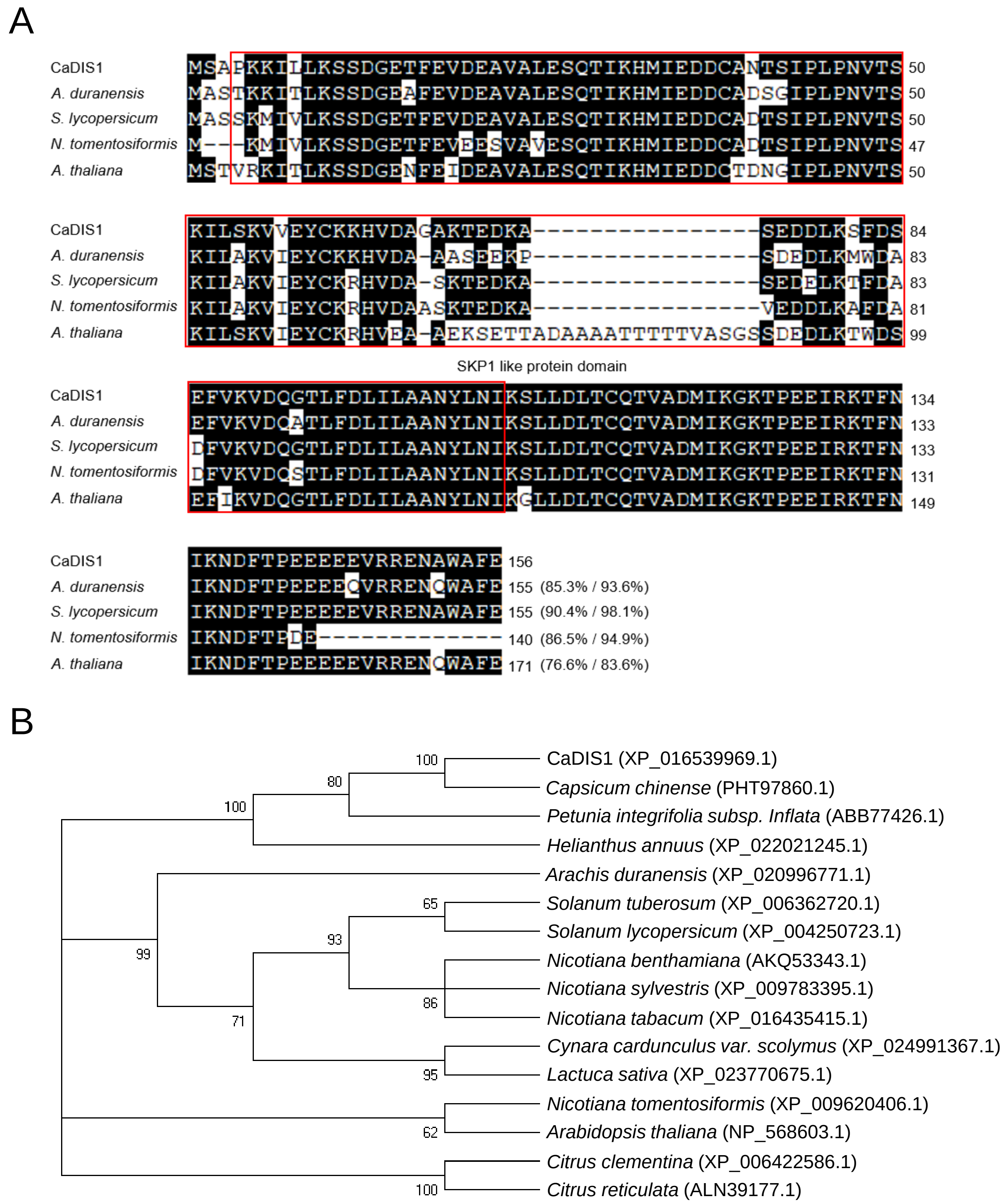

Fig. S2 Lim et al.
